# Catalytic relevance of a quinol anion in biological energy conversion by respiratory complex I

**DOI:** 10.1039/d5sc07500a

**Published:** 2026-01-30

**Authors:** Oleksii Zdorevskyi, Johannes Laukkanen, Vivek Sharma

**Affiliations:** a Department of Physics, University of Helsinki Helsinki Finland oleksii.zdorevskyi@helsinki.fi vivek.sharma@helsinki.fi; b HiLIFE Institute of Biotechnology, University of Helsinki Helsinki Finland

## Abstract

Redox chemistry of quinones is an essential component of life on Earth. In the mitochondrial electron transport chain, the ubiquinone molecule is reduced to ubiquinol by respiratory complex I to drive the synthesis of ATP. By performing both classical and hybrid QM/MM simulations on high-resolution cryo-EM structures, including quantitative free energy calculations, we show that the semiquinone species in complex I is anionic in nature and can be trapped in the active site chamber for its subsequent reduction. Two-electron reduction of ubiquinone yields a metastable ubiquinol anion, which is electrostatically pushed by 15–20 Å towards the exit of the ubiquinone binding chamber to drive the proton pump of complex I. As part of the two-electron reduction of ubiquinone, protonic rearrangements take place in the active site in which a highly conserved histidine converts from its one tautomeric state to another. The combined findings challenge the currently held views on quinone redox chemistry of respiratory complex I and provide a detailed and testable mechanistic picture of the proton-coupled electron transfer reaction at its active site under wild-type and mutant conditions.

## Abstract 2

Quinol anion in respiratory complex I.

## Graphical abstract

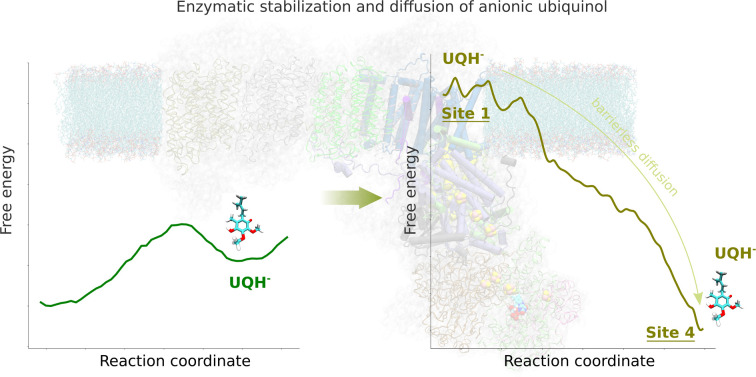

## Introduction

**Fig. 1:**
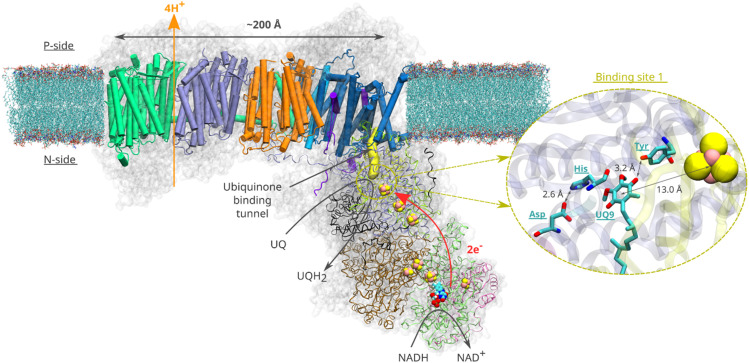
Structural organisation of mitochondrial respiratory complex I from *Yarrowia lipolytica*^25^ (PDB 7O71). Membrane-bound core subunits are shown in cartoon representation, whereas the core subunits of the peripheral arm are shown in ribbon representation. The accessory subunits are represented by a transparent grey surface. A van der Waals representation is used for the redox cofactors of the peripheral arm (iron sulfur clusters and the FMN molecule). The yellow surface represents the UQ chamber – a protein cavity providing access for the UQ species to the redox site (plotted with CAVER (v. 3.0)^52^ using ^49kDa^His95 and ^49kDa^Tyr144 as starting points and a 0.7 Å cutoff). The inset illustrates the binding of UQ at site 1. Spatial orientation of the UQ head group and the adjacent titratable residues are based on the structure^16^ of complex I from *Sus scrofa* (PDB ID: 7V2C).

Quinone molecules are redox-active elements found in all domains of life. By constantly shuttling between their reduced and oxidized forms, they facilitate the transfer of electrons (and protons) to and from various energy converting enzymes.^1^ In the inner membrane of mitochondria, which harbours the electron transport chain, long-tailed ubiquinone (UQ) molecules transfer electrons between membrane-bound redox-active proton pumping enzymes. The first enzyme of this chain, respiratory complex I (Fig. 1), is a NADH:ubiquinone oxidoreductase and couples the oxidation of NADH (*E*_m,7_ ∼ −320 mV) with the two-electron reduction of UQ (*E*_m,7_ ∼ +100 mV). The energy released during UQ reduction is employed to pump protons across the inner mitochondrial membrane^2,3^ (Fig. 1), creating a proton electrochemical gradient, which drives ATP synthesis and active transport.^4^

Despite an extraordinary amount of high-resolution structural data having been reported for complex I for over a decade, its redox-driven proton pumping mechanism still remains elusive and extensively debated^5–8^ (Fig. 1). According to the current consensus (see various proton pumping models discussed in ref. 6), a UQ molecule enters from the membrane milieu to the ∼30 Å long UQ binding chamber (UQ tunnel) and extracts electrons from the terminal FeS cluster N2 (Fig. 1). During tunnel travel, UQ binds to several intervening binding sites within the UQ tunnel (sites 1–5) that were predicted by molecular dynamics (MD) simulations^9–12^ and structurally characterized by cryo-EM.^13,14^ At the deep binding site 1, the UQ head group binds in the vicinity of a conserved His/Asp pair (^49kDa^His95/^49kDa^Asp196 – *Yarrowia lipolytica* complex I numbering) and is stabilised by a hydrogen bond with a conserved tyrosine (^49kDa^Tyr144) sidechain (Fig. 1, inset).^6,15–19^ Bound within the electron transfer distance (<15 Å) from the N2 FeS center (see Fig. 1), the reduction of UQ at site 1 drives electrostatic and conformational transitions^3,6,15,18,20^ that are thought to be important for the coupling mechanism. However, the exact sequence of charge transfer and conformational events remains unknown, resulting in incomplete understanding of the redox-driven proton pumping mechanism of complex I.

Based on the high-resolution cryo-EM structures of complex I in open and closed states,^13,15^ Sazanov and colleagues have proposed a proton pumping mechanism in which an oxidized UQ molecule, tightly bound near the N2 FeS cluster in the closed state, is reduced by two electrons. The two protons are donated by the local proton donors, His/Asp pair and tyrosine, to yield UQH_2_. Computer simulations^18,21^ performed on *Thermus thermophilus* complex I structures obtained from X-ray crystallography^22^ have shown that UQH_2_ can form at site 1 by accepting protons from the His/Asp pair and tyrosine. Classical atomistic MD simulations combined with free energy calculations on bacterial complex I^9–11^ further revealed that UQH_2_ (or menaquinol) binds weakly at site 1 (compared to oxidized UQ) and may diffuse towards the UQ tunnel entrance by binding transiently to other intervening sites within the chamber. However, a question can be raised to what extent the quinol species is protonated. Is it anionic ubiquinol (UQH^−^) or fully protonated charge-neutral ubiquinol (UQH_2_)? Several studies^8,23–26^ have highlighted the importance of anionic quinol species in the proton pumping mechanism of complex I. In the same vein, it is not known if a charge neutral ubisemiquinone or its anionic form (USQ^−^) is stabilized in the redox active site of complex I. Here, we performed classical and hybrid QM/MM (quantum mechanical/molecular mechanical) MD simulations and free energy calculations on the high-resolution cryo-EM structures of mitochondrial complex I to study the protonation and tunnel dynamics of two species of UQ (ubisemiquinone and ubiquinol) that are formed after one- and two-electron transfer, respectively. Our data suggest that the protonation of the ubisemiquinone anion (USQ^−^) is energetically unfavorable and posit anionic ubiquinol (UQH^−^) as a catalytically competent species in the redox catalysis of complex I. We outline a step-by-step mechanistic picture of the UQ reduction and dynamics and provide molecular basis to previously enigmatic site-directed mutagenesis data.

## Results

### Energetics of protonation and dynamics of one electron reduced UQ species

**Fig. 2:**
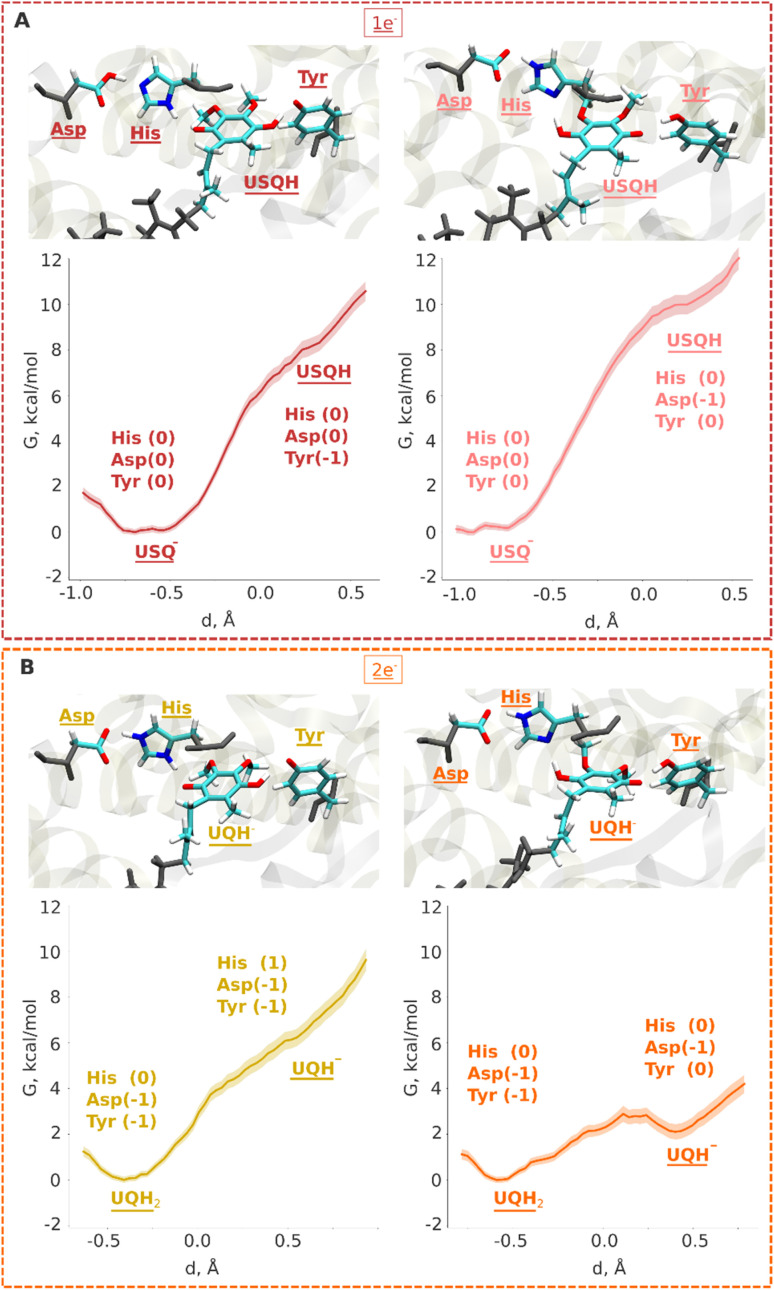
QM/MM umbrella sampling (US) free energy profiles for protonation of ubiquinone in one- (A) and two-electron (B) reduced cases. In all plots, the *y*-axis represents free energy (*G*) in kcal mol^−1^, whereas the *x*-axis displays the reaction coordinate for proton transfer (see the Computational methods). The snapshots display the final states obtained after biased US QM/MM simulations. The neutral ubisemiquinone (USQH) species (at *d* ∼ 0.5 Å) formed after proton transfer from Tyr (A, left) and the His/Asp pair (A, right) is shown in upper panels. The anionic quinol species (UQH^−^) at *d* ∼ 0.5 Å that would form after proton transfer from Tyr (B, left) and His/Asp pair (B, right) is shown in lower panels. The backbone atoms from the MM region are coloured in grey. The whole QM region is shown in Fig. S11.

Electrons are transferred from the terminal N2 cluster to bound UQ one at a time, leading to an inevitable formation of the ubisemiquinone (USQ^−^) radical. Our hybrid (QM/MM) free energy simulations on the high-resolution structure of respiratory complex I (PDB 7V2C, see Computational methods) show that the protonation of USQ^−^ by nearby residues (His/Asp pair and tyrosine) is unfavorable by 8–10 kcal mol^−1^ (Fig. 2A). Spin density analysis reveals that the electron is indeed localized on the UQ headgroup (Fig. S1A), however, a proton transfer does not occur to it, neither from the hydrogen bonded tyrosine nor from the His/Asp pair. At the same time, noticeable protonation and conformational rearrangements take place during QM/MM minimization and simulations. The histidine residue, which is initially modelled as doubly protonated (imidazolium), donates the proton on its ε (epsilon) nitrogen atom to the anionic aspartate in both the fully oxidized and one-electron reduced (USQ^−^) states, thereby rendering both residues charge-neutral with histidine in its δ (delta) nitrogen protonated tautomeric state (Fig. S2A; see also Movie S1). This is in agreement with the classical free energy simulation data that showed charge-neutral states of histidine and aspartate.^17^ Interestingly, our simulation data revealed that histidine can drift from its structural position (as in PDB 7V2C^16^) to form a stable hydrogen bond with the ketonic group of UQ within the timescales of QM/MM simulations (see Movie S1). This conformational rearrangement, which occurs spontaneously in the simulations, brings the His/Asp system into a pose ready for proton donation upon the second electron transfer (see below).

**Fig. 3:**
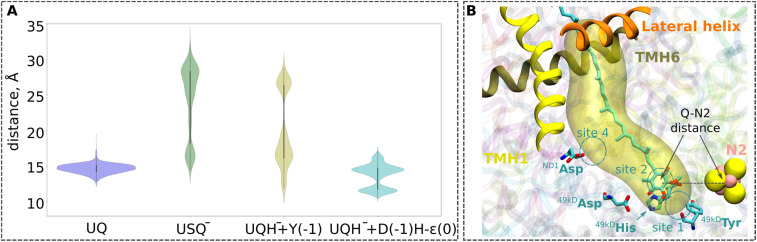
(A) Violin plots for the UQ-N2 distances from the MD simulations (1 µs × 3 replicas) of the high-resolution structure of respiratory complex I from *Yarrowia lipolytica* (PDB ID: 7O71)^25^ with quinone modelled at site 1 (see panel B). The data for UQ are shown in blue, for USQ^−^ in green, and for UQH^−^ in yellow. For the UQH^−^ dataset, ^49kDa^Tyr was additionally deprotonated to model the proton transfer from Tyr to the doubly-reduced quinone (yellow plot). The second UQH^−^ dataset was collected from simulations with an N_ε_ tautomeric state of ^49kDa^His and deprotonated ^49kDa^Asp (cyan plot), modelling proton donation from the His/Asp pair to the doubly-reduced UQ. The time series plots are shown in Fig. S13. The one-letter notations of D, H, and Y correspond to the conserved Asp, His, and Tyr at site 1, respectively (see Fig. 1). (B) Illustration of the distance between the N2 iron–sulfur cluster and the ubiquinone head group (UQ-N2 distance) plotted in panel (A). The distance is measured between the geometrical centers of the quinone head group (atoms C_1_–C_6_), and the N2 iron–sulfur cluster. Three helices (TMH1, TMH6, and the lateral helix) of the ND1 subunit at the entrance to the UQ chamber are shown in ribbons. The cavity is plotted with the CAVER tool (v. 3.0),^52^ taking ^49kDa^Tyr and ^49kDa^His as the reference with a cutoff radius of 0.7 Å.

Even though transient formation of the ubisemiquinone radical is inevitable, analysis of EPR data^27,28^ has raised concerns about the origins and occupancy of the radical species.^6^ The long lifetime of a free radical state such as USQ^−^ in complex I^29^ and its uncontrolled diffusion can lead to proteolipid oxidation and the formation of dangerous ROS. Previous non-equilibrium classical MD simulations showed that a higher amount of force (work) is required to pull a charged USQ^−^ species out of the UQ tunnel, leading to the suggestion that keeping ubiqsemiquinone anionic is a strategy to trap it in the UQ tunnel.^10^ To probe the long-time scale dynamics of oxidised UQ and anionic USQ^−^ in the UQ tunnel, we performed microsecond-long unbiased equilibrium MD simulations on high-resolution structures (PDB 7O71 and 7V2C, see the Computational methods). In contrast to the stable position of oxidised UQ near the N2 FeS center (Fig. 3), the singly reduced UQ (USQ^−^) showed partial unbinding and diffusion towards the UQ binding site 4, closer to the entrance of the UQ tunnel. A similar dynamic behaviour of the singly-reduced UQ species (anionic or neutral USQ) has been observed in the previous MD simulations of different structures of *Y. lipolytica* complex I.^30^ The higher stability of oxidized UQ compared to USQ^−^ is in part due to the stable hydrogen-bonding interactions with neighbouring conserved residues, Tyr and the His/Asp pair (Fig. S3). Notably, the histidine drift observed in small and large QM/MM setups of porcine complex I (see Movie S1) is also reproduced in the completely independent classical atomistic MD simulations of the same complex, thereby consolidating our multiscale modelling and simulation procedure (see Computational methods).

**Fig. 4:**
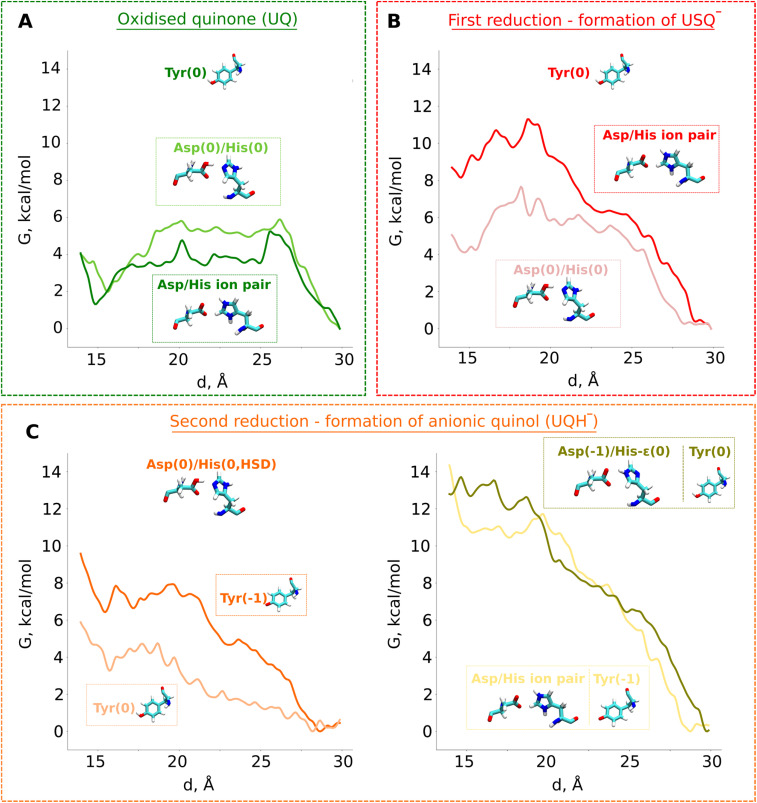
Classical free energy (*G*, kcal mol^−1^) profiles of the propagation of UQ along the binding chamber of respiratory complex I. PMF (free energy) profiles of (A) oxidised ubiquinone (UQ), (B) anionic semiubiquinone (USQ^−^), and (C) anionic ubiquinol (UQH^−^). The *x*-axis corresponds to the distance between the N2 iron–sulfur cluster and the quinone head group (Fig. 3B). The modelled protonation states of ^49kDa^Tyr (denoted as Tyr), ^49kDa^His (denoted as His), and ^49kDa^Asp (denoted as Asp) are shown.

The unbiased MD simulations are not sufficient to fully sample the tunnel dynamics of UQ and USQ^−^. Therefore, to probe the dynamics in a quantitative manner, we performed classical US-based free energy simulations (see Computational methods, Table S4). We find that even though USQ^−^ is in part unstable at site 1 with respect to oxidized UQ (Fig. 4), its diffusion towards the tunnel exit is restricted by a ∼4 kcal mol^−1^ activation energy barrier. This barrier is in part caused by the frequent hydrogen bonds of the USQ^−^ head group with the histidine (Fig. S3B). This will not only lead to the trapping of USQ^−^ at the site near N2 but will also prime it for fast reduction by a second electron transfer, most likely within the nanoseconds of first electron transfer.

Our QM/MM data revealed that protonic rearrangements in the active site lead to stable electrically neutral charge states of histidine and aspartic acid residues (Fig. S2A). To understand the importance of different charge states of these residues on UQ binding, we performed classical free energy calculations in both protonation states of the His/Asp pair by modelling both residues as charged and by modelling both residues as charge-neutral. Contrary to the expected stable binding of anionic USQ^−^ with protonated histidine, we find that USQ^−^ binding with a neutral His/Asp pair is *ca.* 4 kcal mol^−1^ lower in energy than when both Asp and His are in their charged states (Fig. 4B). The unprecedented binding mode, which occurs in both the states of the His/Asp pair (either both neutral or both charged), is in part due to the hydrogen bonds of anionic USQ^−^ with the titratable residues in the deep binding chamber (particularly with histidine; see Fig. S3B). The activation energy barrier of USQ^−^ escape from site 1 to site 4 is found roughly equal in both the charge states suggesting that the anionic USQ^−^ radical can remain trapped in the tunnel, waiting for subsequent electron transfer (Fig. 4B).

### Two-electron reduction and formation of singly- and doubly-protonated ubiquinol

Previous QM cluster models and short time scale QM/MM simulations showed that the two electron reduction results in local protonation of UQ by one or two protons.^18,21^ Here, we performed QM/MM free energy calculations on a high-resolution cryo-EM structure of porcine complex I (PDB 7V2C), which displays a tightly bound UQ, to obtain quantitative estimates of proton transfer dynamics from neighboring proton donors – the His/Asp system and tyrosine (Fig. 1, inset; see Computational methods). We find that the ubiquinol anion (UQH^−^) formed after two-electron reduction is *ca.* 2 kcal mol^−1^ higher in energy than the doubly protonated UQH_2_ state, as long as the first proton transfer occurs from the His/Asp pair (Fig. 2B, right panel). In contrast, proton transfer from the hydrogen bonded tyrosine creates a state with an ubiquinol anion that is much higher in energy (Fig. 2B, left panel, see also Fig. S4), presumably due to the strong electrostatic repulsion between anionic tyrosine and UQH^−^.

To further evaluate that QM/MM free energy calculation results (Fig. 2) do not depend on the size of the QM and MM regions, basis set, density functional, and the choice of the reaction coordinate, we performed additional calculations (see Computational methods, Table S2). Upon two-electron reduction of UQ with a larger QM region (Table S2), a similar free energy profile is obtained (Fig. S5B). Interestingly, when changing the basis set from double-zeta (def2-SVP) to more accurate triple-zeta quality (def2-TZVP) and also with BHLYP (50% exact exchange) density functional (Fig. S6), the barrier separating UQH^−^ and UQH_2_ becomes higher (*ca.* 2 kcal mol^−1^). Such kinetic stabilization of the partially protonated ubiquinol anion (Fig. S6) can have important mechanistic consequences, as discussed below. Even though the energy difference between UQH_2_ and UQH^−^ is only ∼2 kcal mol^−1^ in favour of charge-neutral ubiquinol, this gives equilibrium occupancy of the neutral quinol 10–100 : 1. However, the kinetic barrier of conversion of UQH^−^ to UQH_2_ by proton transfer from conserved tyrosine can be higher and compete with lower activation energy barrier (faster) processes (see the Discussion).

We further scrutinized our unbiased and biased classical and QM/MM MD simulation trajectories of porcine complex I (PDB 7V2C). We find that similar to the oxidized (UQ) and one-electron reduced (USQ^−^) state simulations, a shift in the position of histidine occurs also in the two-electron reduced state. It undergoes a conformational change from its structurally observed position to a direct hydrogen-bonding interaction with the ketone moiety of the UQ head group (Movie S2). However, in stark contrast to UQ and USQ^−^ states, a rapid proton transfer takes place from histidine to doubly-reduced UQ, concomitant with its reprotonation from neutral aspartate (compare Movies S1 and S2), leading to the formation of UQH_2_. Interestingly, this proton relay converts histidine from its one tautomeric state to another (from a proton on δ nitrogen to a proton on ε nitrogen). Therefore, one key difference between one- and two-electron reduced states is that the neutral histidine is in different tautomeric states (N_δ_ and N_ε_ tautomers, respectively); a notion that can be exploited for experimental designs.

### Multi-scale simulations explain the site-directed mutagenesis data

Assuming that the His/Asp pair and tyrosine are the only local proton donors to UQ upon its reduction, one would expect that their mutation to a non-polar residue would yield enzyme completely inactive. However, this is not the case.^6^ Intriguingly, upon mutation of aspartate to asparagine, UQ reduction is not completely suppressed, instead, the enzyme still pumps protons, albeit sluggishly.^18^ Similarly, mutation of tyrosine to phenylalanine renders the enzyme catalytically active with proton pumping.^19,31^ Point mutation of histidine to arginine^31^ also yields an enzyme that is still partially active. These data clearly show that single point mutation of putative proton donors to UQ is well tolerated and that upon two-electron reduction of UQ, a proton must be transferred from the available donor (either tyrosine or the Asp/His pair). Not surprisingly in our mutant QM/MM MD simulations, we observed that reduction-coupled proton transfer occurs either from the Asp/His system or tyrosine leading to the formation of anionic ubiquinol (Fig. S2B and C). We next investigated the influence of point mutations by calculating the electron affinities of WT and mutant systems (see Computational methods). Notably, the data suggest that the first electron transfer is energetically comparable for both WT and mutants (Fig. S7); however, for the second electron transfer (which is always coupled with proton transfer), the electron affinities of mutants are in part lower, commensurate with their lower catalytic activity and sluggish proton pumping.^6^ These data suggest that UQH^−^ is most likely the species that forms during two-electron reduction and drives the catalysis of mutated complex I (see the Discussion).

### Tunnel dynamics of anionic ubiquinol

To study the long-time scale behaviour of UQH^−^ species, we performed classical MD simulations in two different states representing proton donation either from tyrosine or from the His/Asp pair (Fig. 3A). The high energy state representing anionic tyrosine (Fig. 2B, left panel) showed occupancy of anionic quinol at UQ binding sites 1 and 4 (Fig. 3A, yellow). However, no such diffusion of UQH^−^ is observed in the limited time scales of unbiased MD simulations when proton transfer occurs from the His/Asp system instead of tyrosine (Fig. 3A, cyan). In order to trigger the departure of UQH^−^ from site 1 and probe its energetics, we performed classical US MD simulations on several different states (Table S4 and Fig. S8). We find that UQH^−^ formed after single proton transfer is almost equally strongly repelled either by anionic tyrosine or by the negatively charged His/Asp pair (Fig. 4C, right panel; light yellow and olive-green traces, respectively). The obtained potential of mean force (PMF) profiles display almost barrierless UQH^−^ exit from site 1 towards site 4, in stark contrast to UQ and USQ^−^ cases representing oxidized and one-electron reduced UQ, respectively, where diffusion is impeded by kinetic barriers (Fig. 4). In agreement with the data shown in Fig. 2 and S2A, the PMF profile in states with anionic tyrosine show 5–6 kcal mol^−1^ stabilization of neutral charge states of histidine and aspartate than their charged forms (Fig. 4 and S8). As alluded to above, electrostatic repulsion between UQH^−^ and anionic tyrosine is likely the reason for the high energy of the state (Fig. 2), we thus simulated the exit of UQH^−^ from site 1 by modelling the neutral charge state of the tyrosine residue (Fig. 4C). The lowering of the energy of UQH^−^ binding near the N2 center by *ca.* 3 kcal mol^−1^ points to the key role of electrostatic interaction in driving anionic ubiquinol away from site 1. A much stronger energetic stabilization is observed upon neutralization of either histidine or aspartate of the His/Asp pair (net charge change from −1 to 0) with tyrosine modelled as neutral (Fig. S8), further highlighting the importance of electrostatics in driving UQH^−^ away from site 1 and towards site 4, and indicating that His/Asp pair deprotonation may provide a stronger driving force for UQH^−^ exit than anionic tyrosine (see the Discussion).

The above presented US data on the tunnel dynamics of different UQ species are based on the high resolution cryo-EM structure of complex I from *Yarrowia lipolytica* (PDB 7O71), which does not bind a UQ molecule at the site near the N2 FeS cluster. Even though MD simulation data support the notion that an oxidized UQ can bind at site 1, and relatively strongly compared to the anionic ubiquinol (UQH^−^, Fig. 4), there is a possibility that the observed weak binding of the latter species is in part driven by the protein conformation resolved in a state that prevents stabilization of the substrate. Therefore, we decided to probe the binding and dynamics of oxidized UQ and UQH^−^ based on the high-resolution cryo-EM structure of complex I from *Sus scrofa* (PDB 7V2C) in which a UQ molecule is structurally resolved at the site close to the N2 FeS cluster (site 1). Our US data show that an oxidized UQ molecule can bind at site 1 with a relatively large (4–7 kcal mol^−1^) activation energy barrier towards the tunnel entrance in contrast to what is observed in the US simulations based on *Yarrowia* complex I (Fig. S9). A higher activation barrier would prevent the release of oxidized UQ towards tunnel entrance and is in agreement with the structural data on porcine complex I that display a tightly bound UQ molecule. When we probed the energetics of UQH^−^ species, in close analogy to the energetics of UQH^−^ obtained from *Yarrowia* complex I simulations, we find that anionic ubiquinol binds weakly in comparison to the oxidized UQ state, also in the porcine complex I simulations. However, the diffusion of UQH^−^ towards tunnel entrance is impeded by an energy barrier of *ca.* 5 kcal mol^−1^, which is likely caused by an alternative conformation of the TMH5–6 loop (see olive green profile in Fig. S9), because the other UQ-tunnel facing loop, β1–β2 from the 49 kD subunit, is in a similar conformation in the two structures. Therefore, we re-modeled the TMH5–6 loop of porcine complex I to the conformation observed in the *Yarrowia* complex I (see the Computational methods) and observed that the energy barrier dropped from ∼5 kcal mol^−1^ to ∼2 kcal mol^−1^ (see grey profile in Fig. S9), thereby bringing the UQH^−^ PMF profiles in two complexes comparable to each other. We emphasize that even though the results obtained on porcine complex I after loop remodeling improved the energetics of UQH^−^ diffusion, additional factors such as charge states of residues can further influence the results. We also note that in the biased MD simulation approaches such as US, it is extremely challenging to achieve full equilibration of the degrees of freedom outside the biased reaction coordinates. Therefore, a complete description of dynamics and associated energetics can be difficult to achieve. Nevertheless, our extensive US data on the remodeled porcine complex I structure shows that the diffusion of UQH^−^ in the UQ tunnel correlates with the loop movements,^3^ and resembles the UQH^−^ dynamics observed in a simulation of a complex from another species in a different catalytic state.^25^

We also modelled the UQH_2_ species in *Sus scrofa* complex I and find that, similarly to UQH^−^, it binds unfavourably at site 1 (see yellow profile in Fig. S9). Even though both species show an energetically downhill trend towards the UQ tunnel entrance, UQH_2_ shows somewhat barrierless diffusion. Previously observed local energy minimum for UQH_2_ (ref. 10) or for fully-reduced fully-protonated menaquinol^11^ are not observed in the current simulations, most likely due to the differences in the 3D structures used in these studies (see the Discussion).

## Discussion

**Fig. 5:**
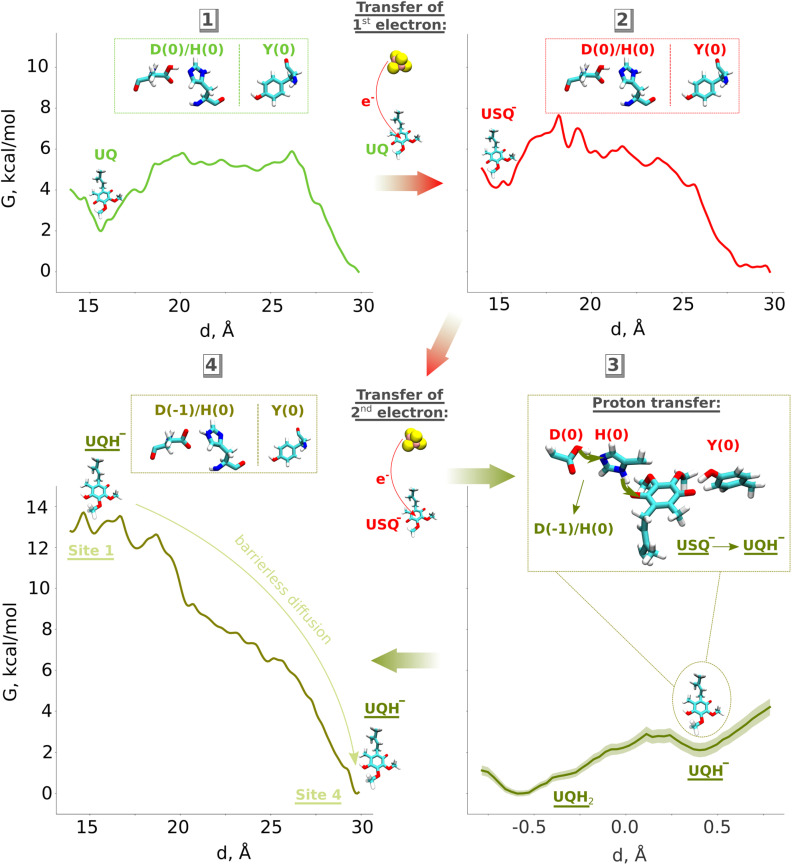
Proposed mechanism of ubiquinone reduction and dynamics in respiratory complex I. Oxidised UQ (top left) gets an electron from the N2 iron–sulfur cluster to become anionic ubisemiquinone (USQ^−^, top right). Upon accepting the second electron from N2 (bottom left), a proton is transferred from the His/Asp pair leading to the formation of anionic ubiquinol (UQH^−^). The electrostatic repulsion by anionic His/Asp drives the diffusion of UQH^−^ towards site 4 of the UQ binding chamber. D, H and Y denote the Asp, His, and Tyr residues at site 1, respectively (see Fig. 1).

Based on our classical and QM/MM computations, we propose a stepwise mechanism of UQ reduction and its dynamics in the UQ tunnel. The proposed mechanism is in agreement with our recently developed model on the coupling mechanism of respiratory complex I,^6^ which we schematically illustrate in Fig. 5. The oxidised UQ binds at site 1 within the electron transfer distance to the N2 FeS cluster. A kinetic barrier, the height of which partly depends on the conformation of the conserved TMH5–6 loop of the ND1 subunit, prevents the diffusion of UQ to site 4 (Fig. 5, top left panel). At this stage, both tyrosine and the His/Asp pair are charge-neutral, with histidine (N_δ_ tautomer) and aspartic acid each holding one proton. Upon the first electron transfer to UQ, the semiquinone radical species is formed. The protonation of anionic semiquinone either from tyrosine or from the His/Asp pair is unfavourable and it remains anionic in nature, which could be experimentally tested by EPR approaches. The binding of USQ^−^ at site 1 is less favorable compared to oxidized UQ, but the kinetic barrier towards site 4 can prevent its escape (Fig. 5, top right panel) thereby enhancing the probability of its reduction by the second electron. The second electron transfer occurs concomitantly with a proton transfer from the His/Asp pair (not from Tyr), where a proton relay converts histidine from its one tautomeric state to another (from N_δ_ to N_ε_ tautomer; see also Fig. 5, bottom right panel), a notion that may be investigated with the NMR experiments. The anionic ubiquinol formed (see also ref. 23) is highly unstable at the site and is strongly repelled by the now anionic His/Asp pair. At this stage, either deprotonation of tyrosine occurs to form UQH_2_ or UQH^−^ departs. The nearly isoenergetic states (UQH^−^ and UQH_2_) suggest that the reaction may proceed by involving either of the two species (see more below). There is a strong driving force for the diffusion of UQH^−^ away from site 1 and towards site 4 (Fig. 5, bottom left panel; see also Fig. S9). After reaching site 4, the anionic quinol drives the proton pump of respiratory complex I by a proton injection-driven proton pumping mechanism^6^ (*cf.* also ref. 5 and 7). Our results posit that both the ubisemiquinone radical and ubiquinol are likely anionic in nature and the occupancy of the latter is likely enhanced in the mutants of the active site residues (histidine, tyrosine and aspartate). Thus, it would be desirable to look for spectroscopic signals in the mutated enzyme to strengthen the functional importance of anionic ubiquinol.

The possibility that anionic quinol (UQH^−^) is an important catalytic intermediate is in agreement with the recent findings,^8^ where, based on theoretical and DFT-based modelling, it was shown that single protonation of doubly-reduced singly protonated ubiquinone (*i.e.* anionic quinol) drives the proton pump of complex I. It was also suggested that one-electron reduced state semiquinone is charge neutral.^8^ However, our QM/MM free energy calculations suggest that semiquinone species is anionic in nature; that is the first electron transfer to UQ from N2 is not coupled to local protonation. The previous classical MD simulation data on *Yarrowia* complex I structures revealed that neutral semiquinone is much more likely to diffuse in the UQ tunnel.^30^ The higher mobility of neutral semiquinone would enhance the possibility of its redox reactions with the protein and lipid molecules, thereby leading to undesirable ROS formation. Therefore, the stabilization of anionic semiquinone might be an enzymatic strategy to keep the levels of ROS load low.

Our QM/MM free energy simulations show that the doubly reduced and doubly protonated UQH_2_ species is somewhat lower in energy than the anionic UQH^−^. At equilibrium, this can yield a higher occupancy of UQH_2_, which has been suggested to be the key species that is formed after two-electron reduction and local protonation by the His/Asp pair and tyrosine (see the Introduction). Though, it is possible that under turnover conditions the equilibrium is shifted in favour of UQH^−^. We also note that the ∼2 kcal mol^−1^ energy difference between UQH_2_ and UQH^−^ is within the uncertainties associated with the multiscale computational modelling. Additional factors such as the inclusion of the N2 FeS cluster and its surroundings in the QM region can also perturb the [UQH_2_]:[UQH^−^] ratio due to the short- and long-range effects. Such aspects are known to play a key role complex I mechanism.^20,32^ The QM/MM PMF profiles show that the two states (UQH_2_ and UQH^−^) can be separated by a kinetic barrier, which may trap the conversion of UQH^−^ to UQH_2_. Since UQH^−^ in porcine complex I is strongly displaced from site 1 at *x* ∼ 12 Å to the region in the Q tunnel around *x* ∼ 15 Å (see Fig. S9, energetically downhill profile), a longer distance of its headgroup from tyrosine (12–14 Å) will make the transfer of proton(s) energetically less favourable. It is known that proton transfer through longer pathways (water wires, *e.g.*) is energetically more demanding than through shorter paths.^32^ This can make the protonation of UQH^−^ slower than its departure from site 1 to site 4 (*x* ∼ 25 Å). Indeed, classical umbrella sampling simulations on two different complex I structures support the view and show that UQH^−^ formed at site 1 is highly unstable and its departure towards site 4 can occur with low activation energy barriers, especially when coupled to conformational dynamics of UQ tunnel facing loops.

Earlier unbiased MD simulations demonstrated that UQH_2_ diffusion is slower in comparison to oxidized UQ, and it is likely to be trapped in the vicinity of sites 1 and 2 (ref. 9) (see also ref. 30). Free energy simulations on bacterial complex I^10,11^ also revealed energy barriers of *ca.* 2–3 kcal mol^−1^ for the diffusion of UQH_2_ from site 1 to site 4. The activation energy barriers for UQH^−^ diffusion are found to be in the similar range when conformational changes of UQ tunnel facing loops are accounted for. However, for UQH_2_ in mitochondrial complex I from *Sus scrofa*, a local energy minimum is not observed (Fig. S9), and UQH_2_ once formed after two-electron reduction and local protonation from the His/Asp pair and tyrosine, would diffuse out to site 4 with no large activation energy barriers. This is in agreement with the earlier mechanistic suggestions^5,7,10^ and also indicates that the porcine complex I conformation is capable of trapping oxidized UQ at site 1, whereas UQH_2_ (and also UQH^−^) binds much more weakly. Further experimental and computational studies are required to reveal the identity of the true catalytic intermediate (UQH^−^ or UQH_2_). However, our current results suggest that anionic ubiquinol can be considered and examined as a potential catalytic intermediate in the proton pumping mechanism of complex I. Our calculations also support the emerging view that active site tyrosine is not a primary proton donor to UQ upon its reduction but assists in trapping it to the binding site.^6^

It is important to emphasize that even though MD simulations (also QM/MM type) provide dynamical insights on protein structures, sufficient sampling of conformational space remains a bottleneck. Even with the enhanced sampling approaches,^33^ which are computationally intensive, care must be taken on selecting the 3D structures to initiate simulations, especially when answering specific mechanistic questions. By performing computer simulations on different protein structures, a holistic functional picture may emerge in some cases. However, because exhaustive sampling of all degrees of freedom is difficult to achieve, a common mechanistic denominator may not be found. In the present work, we observed that the US-based free energy profiles of the UQ molecule in respiratory complex I may differ depending on which 3D structure of the protein is employed. Starting with the free energy profiles of oxidized UQ in the UQ tunnel, we find that the current results on mitochondrial complexes are in reasonable agreement with the previous free energy estimates on bacterial complexes (see the Introduction). However, for UQH^−^ and UQH_2_ species, some differences are observed. UQH^−^ shows nearly a barrierless transfer from site 1 towards site 4 in the *Yarrowia* complex I structure. However, it is found to be impeded by an activation energy barrier in simulations on *Sus scrofa* complex I. Among other factors, one of the reasons is the different conformation of the conserved TMH5–6 loop facing the UQ tunnel in the two structures. When the porcine complex I TMH5–6 loop is remodeled in *Yarrowia* complex I conformation, an appropriate change in the PMF profile of UQH^−^ is observed, highlighting that the simulations can capture the modelled structural changes and reflect in energetics. Nevertheless, a complete agreement may be hard to achieve due to several subtle structural differences, including the approximate and fixed charge state description of the titratable amino acid residues. The activation barrier heights can also be challenging to obtain, especially when the protein structures involve conformationally mobile loops, which is the case with the UQ tunnel. The UQ cavity is lined up with several dynamic loops carrying conserved titratable residues. Even with the application of state-of-the-art enhanced sampling methods, the energetic estimates can sometimes be semiquantitative in nature at best. The accuracy is also further affected by the limited resolution of the 3D structure, low accuracy of sidechain conformation of acidic residues in cryo-EM maps, underestimated hydration, *etc.*^34^

If differences were observed in free energy calculations of complex I from two species, some converging results were also obtained from classical and QM/MM MD simulations of *Sus scrofa* complex I. The histidine residue of the conserved His/Asp pair is found to rearrange from its structural position in both independent setups, thereby consolidating the multiscale computational strategy applied here. The QM/MM results also highlighted the changes in the tautomeric state of histidine depending on the redox state of UQ. Whether these results can be extended to other high-resolution complex I structures is difficult to generalize. Nevertheless, our work clearly highlights advantages and disadvantages of performing computer simulations on variable structural data from different organisms obtained under different experimental conditions (catalytic states).

The cryo-EM structures of respiratory complex I can attain several distinct conformational states, some of which have been labelled as open and closed.^13,15^ Based on the analysis of several high-resolution cryo-EM structures, it has been suggested that these states correspond to catalytic intermediates. A UQ molecule binds at site 1 near the N2 center in the closed state and is reduced and protonated to UQH_2_.^5^ The departure of UQH_2_ towards the lipid membrane *via* the UQ tunnel entrance is coupled to the closed-to-open state transition. The high-resolution cryo-EM structure of porcine complex I (PDB 7V2C), which tightly binds a UQ molecule, is likely resolved in the closed-like state. On the other hand, the *Yarrowia lipolytica* complex I structure (PDB 7O71), which does not bind UQ molecule at site 1 (but likely at site 4, see *e.g.* ref. 14), shows a mixed (open/closed) arrangement of the tunnel-facing loops (*e.g.* β1–β2 loop is in a closed-like conformation). Similar mixed states, where both open/closed-like conformations of key functional loops are observed, have been reported earlier.^15,35^ When we modelled the UQH^−^ molecule in the UQ chamber of porcine complex I, it is found to bind weakly at site 1 in contrast to oxidized UQ. However, its diffusion towards the UQ tunnel entrance is blocked by a kinetic barrier, which is likely caused (or at least in part) by the closed-like conformation of the TMH5–6 loop. This loop when modelled in an alternative conformation resolved in *Yarrowia* complex I, the energy barrier towards the UQ tunnel exit reduces. Even though this hybrid *in silico* model, prepared by TMH5–6 loop remodeling, may not resemble to any functional state, the data do suggest that UQH^−^ exit is likely to be coupled to the closed-to-open transition. However, in contrast, the departure of UQH_2_ towards the tunnel entrance in the native porcine complex I structure is found to be partially barrierless and therefore could occur already in the closed-like states of the protein complex. Further experimental and computational work is required to prove the identity of the catalytic intermediate (UQH^−^ or UQH_2_) and their involvement in open/closed (active/deactive) transitions of the complex. We note that even though there is some convergence in the field on the structures of open and closed (active and deactive) catalytic states, several issues persist.^36,37^ These and other uncertainties in cryo-EM models pose a multiscale challenge for computational methods. A more integrated approach bridging simulations with experiments is required in the future to provide a full atomistic picture of these functional transitions.

## Computational methods

### Classical MD simulations

To study the long-timescale UQ dynamics in the UQ tunnel, we performed classical atomistic MD simulations of the high-resolution (2.4 Å) cryo-EM structure of respiratory complex I from *Yarrowia lipolytica*^25^ (PDBID 7O71). The model system comprised 8 core subunits: ND3, ND1, 49 kDa (excluding long terminus with residue IDs from 29 to 79), 30 kDa, PSST, TYKY, ND4L, and ND6, as well as 10 accessory subunits: 39 kDa, B13, B14.5a, B17.2 (excluding residue IDs 124–137), B14, PGIV, B16.6, 15 kDa, B9, and MWFE (Fig. S10). The protein system was embedded into a lipid bilayer consisting of 50% POPC, 35% POPE and 15% cardiolipin, with a periodic water/ion box of 145 Å × 160 Å × 200 Å and a Na^+^/Cl^−^ salt concentration of 150 mM. The total simulation model comprised ∼365 000 atoms. A truncated model of *Yarrowia* complex I is constructed to keep the system size tractable and for enhanced simulation sampling (see free energy simulations below). Similar truncated model systems of mitochondrial and bacterial complexes have been simulated in the past, yielding novel functional and mechanistic insights, with simulation trajectories showing stable behaviour.^9,30,38^

As no quinone molecule was resolved in the cryo-EM structure of native *Yarrowia* complex I (PDBID 7O71), we modelled UQ (nine isoprene units) species in the deep binding chamber by aligning the UQ coordinates taken from the complex I structure (PDBID 7V2C).^16^ Previously, microsecond-long atomistic MD simulations have been performed on the native state *Y. lipolytica* complex I,^6,25^ but without a modelled UQ in its active site. Our current MD simulations reveal that an oxidized UQ can stably bind in the deep UQ binding site 1 and form hydrogen bonding interactions with the conserved active site residues (Fig. S3). Additionally, we simulated the full mitochondrial complex I structure from *Sus scrofa*^16^ that binds a UQ molecule (ten isoprene units). Multiple simulation replicates of this setup (Table S4, setup 2) show that UQ stably binds in the deep UQ binding site 1 (Fig. S13C) with hydrogen bonds to active site residues (Fig. S3E). A small-scale model of porcine complex I (PDB 7V2C), consisting of the same core subunits as the truncated model of *Yarrowia* complex I, was also constructed for free energy simulations (see below).

The CHARMM36 force field was used for protein,^39^ lipids^40^ and solvent water (TIP3P) and ions.^41^ We used different charge states of the UQ ligand: neutral (oxidised) state (UQ), anionic ubisemiquinone (USQ^−^, charge −1), and anionic ubiquinol (UQH^−^, charge −1). Parameters for different UQ species were taken from ref. 9, 42 and 43. With USQ^−^, we modelled the first step of quinone reduction, and with UQH^−^ the reduction by two electrons and local protonation (either from ^49kDa^Tyr144 or from the ^49kDa^His95/^49kDa^Asp196 pair). The system was first minimised for 1000 steps using the conjugate-gradient algorithm, as implemented in NAMD (v. 2.14).^44^ During the minimisation procedure, protein heavy atoms, the ubiquinone ring, and the lipid phosphorous atoms were harmonically restrained to their structural positions with the potential *U* = *k* × (*x* − *x*_0_)^2^ and with a force constant of 4.14 × 10^4^ kJ (mol^−1^ nm^−2^). After that, we performed energy minimisation in GROMACS (v. 2021.2)^45^ using the steepest-descent algorithm with a force tolerance of 250 kJ (mol^−1^ nm^−1^), followed by a 10-ns equilibration in the NPT ensemble with the same constraints (potential *U* = *k*/2 × (*x* − *x*_0_)^2^). Simulation replicas 2 and 3 were generated by extending the above equilibration procedure to 11 ns and 12 ns, respectively, therefore yielding a different starting conformation. The equilibration stage was concluded by a 10-ns NPT simulation with harmonic restraints on protein backbone atoms (20 000 kJ (mol^−1^ nm^−2^)). In the production run, all atoms were kept free. All classical MD simulations were performed with GROMACS (v. 2021.2) software^45^ using the V-rescale thermostat^46^ and Berendsen barostat,^47^ and a 2-fs time step was achieved with LINCS.^48^ Hydrogen bonds were analysed with the Hbonds VMD plugin^49^ with the distance and angle cutoffs of 3.5 Å and 30°, respectively.

### Classical free energy simulations

Classical umbrella sampling (US) simulations were performed using the 330th-ns (*Yarrowia lipolytica* complex I) and 1107th-ns (*Sus scrofa* complex I) snapshots of the unbiased MD production run with bound UQ (see Table S4, setups 1.1 and 2.1, respectively). In order to avoid convergence problems, we mutated UQ with 9 isoprenoid units to a UQ with a single isoprenoid unit, and then “pulled” the quinone from site 1 to site 4 with a velocity of 10^−7^ Å fs^−1^ and a force constant of 10^3^ kJ (mol^−1^ nm^−2^). The reaction coordinate was represented by the distance between the geometrical centers of the N2 iron–sulfur cluster and the quinone head group ring (Fig. 3B). Different umbrella sampling windows were generated by extracting the snapshots from the “pulling” run with a 0.5 Å stride between the reference values of the reaction coordinate. In order to stabilize the actual Q-N2 distance to the reference distance, each window was equilibrated for 10 ns with a larger force constant of the biasing potential (10^4^ kJ (mol^−1^ nm^−2^)). Then, we carried out a 20-ns equilibration with the lower force constant (10^3^ kJ (mol^−1^ nm^−2^)). The production simulation (NVT, Gromacs 2024 beta) used for plotting free energy profiles was performed for 60 ns with a force constant of 10^3^ kJ (mol^−1^ nm^−2^). The histogram unbiasing procedure was implemented using the WHAM analysis tool (v. 2.0.10).^50^ US setups for quinone in different charge (redox and protonation) states (*e.g.* USQ^−^ and UQH^−^) were created from the initial simulation snapshots of the oxidised UQ state by converting quinone to the respective charge state in each of the simulation windows. The convergence plots of the PMF profiles for different time series are shown in Fig. S14. The bootstrapping errors estimated with a 20-ps correlation time were sufficiently small (∼0.02–0.03 kcal mol^−1^) and are thus not shown on the free energy profiles.

In addition to the simulations on native protein conformations, we also carried out US free energy simulations on the *Sus scrofa* complex I structure (PDB 7V2C) by remodeling the TMH5–6 loop of the ND1 subunit (residues 199–210) to the conformation observed in *Yarrowia lipolytica* complex I (PDB 7O71, see Table S4, setup 3.3). The homology modelling was performed with the MODELLER tool.^51^

Figures were prepared with VMD (v. 1.9.4),^49^ and cavities were plotted with the CAVER (v. 3.0) software tool for protein analysis and visualisation.^52^

### Hybrid QM/MM simulations

Hybrid quantum-mechanical/molecular-mechanical (QM/MM) MD simulations were performed on the high-resolution structure of mitochondrial complex I from *Sus scrofa*,^16^ where the UQ ligand has been fully resolved in the UQ tunnel (Fig. S11, inset). Six core subunits were included in the model system: ND3, ND1, 49 kDa, 30 kDa, PSST, and TYKY. To eliminate the large spatial fluctuations of the long N-terminus of the 49 kDa subunit, residues 34 to 78 were excluded from the model system.

To study the protonation dynamics of the oxidized and reduced UQ species, we first carried out unbiased QM/MM MD simulations of the quinone ligand structurally resolved at site 1, forming a direct hydrogen bond with the highly conserved Tyr141 of the 49 kDa subunit (Fig. 1, inset). The QM region comprised protein sidechains surrounding the UQ head group (Table S1). For QM/MM boundary, protein amino acids were pruned at the C_α_–C_β_ bond, while the QM/MM bond for Q_9_ was created between the C_11_ and C_12_ atoms. Linking hydrogens were placed along the QM/MM bonds, as implemented in the NAMD QM/MM module.^53^ Hydrogen atoms were added to the structural conformation using the PSFGEN plugin for VMD,^49^ and the protonation states of the titratable residues were derived from the p*K*_a_ calculations performed with PROPKA software.^54^ The system was embedded into a lipid bilayer containing 50% POPC, 36% POPE, and 14% cardiolipin, and immersed into a water-ion box with the dimensions 110 × 124 × 125 Å^3^, and with the 150 mM concentration of Na^+^/Cl^−^ salt. In total, the simulation system comprised around 211 000 atoms.

The simulations were carried out with the NAMD molecular dynamics package (v. 2.14)^44,53^ in combination with the ORCA quantum chemistry program (v. 5.0.3).^55–57^ The MM part was treated with the CHARMM36 force field,^39^ where the non-bonded interactions cutoff was set to 12 Å, with switching and pairlist distances equal to 10 Å and 14 Å, respectively. The system was classically minimised for 1000 steps with the conjugate-gradient algorithm, followed by a 10-ns classical MD equilibration in the NPT ensemble with harmonic restraints on all heavy atoms of protein and quinone (with a force constant of 99.0 kcal (mol^−1^ Å^−2^)). In order to further “relax” the system on its free energy landscape, an additional classical 10-ns equilibration stage with constraints on the protein backbone (force constant of 20 000 kJ (mol^−1^ nm^−2^)) was performed in certain setups (Table S2). This was followed by the QM/MM minimisation for 200 steps and the unbiased QM/MM MD run. Temperature was maintained at 310 K using a Langevin thermostat,^58^ and the pressure control was implemented with the Langevin piston barostat.^59,60^ The QM region was described by density functional theory (DFT) with the hybrid B3LYP functional^61–63^ and the def2-SVP basis set.^64^ The DFT-D3 dispersion correction^65^ was used to account for the long-range London dispersion forces, and the energy tolerance for the self-consistent field procedure was set to 10^−8^ au. The additive electrostatic embedding scheme^53^ was used to impart Coulomb interactions between the QM and the MM regions.

### QM/MM free energy simulations

To probe the energetics of proton transfer, we carried out free energy umbrella sampling simulations. To facilitate the proton transfer reaction, the reaction coordinate was chosen as a linear combination of the distances forming the proton pathway (Fig. S12):*d* = *r*_1_ − *r*_2_.

The first simulation window was created by applying a harmonic potential to this reaction coordinate, restraining it to its equilibrium distance:*U*(*r*) = *k*/2 × (*r* − *r*_0_)^2^,with the force constant *k* = 100 kcal (mol^−1^ Å^−2^). After that, the equilibrium distance *r*_0_ was moved by 0.02 Å every step to create subsequent windows with a stride of 0.2 Å. The bias to the reaction coordinate was introduced with the flexible Colvars module,^66^ implemented in NAMD. Each window was simulated for 3.8 ps in total, where the last 2 ps were taken for the calculations of the potential of mean force (PMF) profiles. These calculations were carried out using the weighted histogram analysis method with the WHAM (v. 2.0.10) analysis tool.^50^ The bootstrapping errors were calculated using the correlation time of 20 fs and are depicted as a shaded area on the free energy profiles.

To study the energetics of protonation of anionic USQ^−^, a biased proton transfer was simulated either from neutral tyrosine or from the neutral His/Asp pair to yield a neutral ubisemiquinone state with anionic tyrosine or the anionic His/Asp pair, respectively. Similarly, to track the energetics of protonation of doubly-reduced ubiquinone, a biased proton transfer was simulated from UQH_2_ to anionic tyrosine or to the anionic His/Asp pair, yielding states corresponding to protonation of UQ from the His/Asp pair or tyrosine, respectively, within our mechanistic model. To further test the choice of the reaction coordinate, we performed additional US simulations by including the linear combination of His–Asp distances to the reaction coordinate for the UQH_2_-His proton transfer in a two-electron reduced state (Fig. S15, top inset). We show that the different reaction coordinate does not induce the formation of a metastable UQH^−^/His-ε(0)/Asp(0)/Tyr(−1) state (Fig. S15), coherent with our result for the UQH_2_-His reaction coordinate (Fig. 2). Additional QM/MM free energy simulations were performed with BHLYP^63,67^ density functional, full structure of *Sus scrofa* complex I in membrane/solvent, as well by modelling the electronic structure of anionic quinol as an open shell singlet (Table S2 and Fig. S6).

We performed a total of ∼45 µs of classical and ∼400 ps of QM/MM MD simulations.

### Electron affinity calculations

The electron affinity for the first electron (Fig. S7) was calculated as the energy difference between oxidised ubiquinone (*E*_UQ_) and ubisemiquinone (*E*_USQ_):*E*_1e_ = *E*_UQ_ − *E*_USQ_.

Similarly, the electron affinity for the second electron was derived as the energy difference between ubisemiquinone (*E*_USQ_) and ubiquinol (*E*_UQH_):*E*_2e_ = *E*_USQ_ − *E*_UQH_.

Energies were calculated as averages between 501st and 1500th fs of unbiased MD. Standard deviations for electron affinities *σ*_1e_, *σ*_2e_ were calculated as the square-root of the sum of squares of deviations of the respective individual distributions:
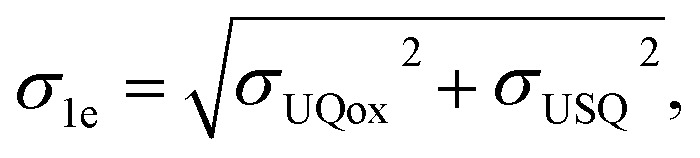

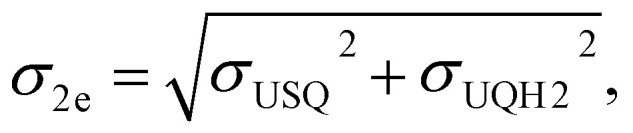
where *σ*_UQox_, *σ*_USQ_, and *σ*_UQH2_ are the standard deviations on the QM/MM energy distributions in the zero-, one-, and two-electron reduced states, respectively.

## Author contributions

OZ performed QM/MM MD simulations, classical MD simulations, and free energy calculations; analysed the data; prepared the figures; co-supervised JL; and wrote the initial draft of the manuscript. JL performed QM/MM MD simulations, classical MD simulations, and free energy calculations and analysed the data. VS designed and supervised the research, analysed the data, and wrote and refined the text.

## Conflicts of interest

There are no conflicts of interest to declare.

## Supplementary Material

SC-OLF-D5SC07500A-s001

SC-OLF-D5SC07500A-s002

SC-OLF-D5SC07500A-s003

SC-OLF-D5SC07500A-s004

## Data Availability

The data supporting this article have been included as part of the supplementary information (SI). Supplementary information is available. See DOI: https://doi.org/10.1039/d5sc07500a.
