# Supplementary material for: Catalytic relevance of a quinol anion in biological energy conversion by respiratory complex I
Source: Chem Sci. 2026 Jan 30;17(13):6688–701. doi: 10.1039/d5sc07500a (PMC12890310; doi:10.1039/d5sc07500a)
Supplement: SC-017-D5SC07500A-s004 [file SC-017-D5SC07500A-s004.pdf]

Supplementary Information (SI) for Chemical Science.
This journal is © The Royal Society of Chemistry 2026

Supplementary Information

Catalytic relevance of quinol anion in biological energy conversion by respiratory complex I

Oleksii Zdorevskyi[1,*], Johannes Laukkanen[1], Vivek Sharma[1,2,*]

[1]Department of Physics, University of Helsinki, Helsinki, Finland
[2]HiLIFE Institute of Biotechnology, University of Helsinki, Helsinki, Finland

Correspondence to;
Oleksii Zdorevskyi (oleksii.zdorevskyi@helsinki.fi)
Vivek Sharma (vivek.sharma@helsinki.fi)

Supplementary note

Additional visual analysis of long-timescale classical MD simulations of *Yarrowia* complex I showed a novel stable conformational arrangement of hydrogen bonding between histidine residue and methoxy groups of UQ (ca. 25% of simulation time, Fig. S3). To probe the protonation dynamics in this conformational state, we initiated additional QM/MM setups starting from the classical MD snapshot in which histidine is stabilized in hydrogen-bonding with the methoxy groups of UQ (see Table S2). In QM/MM MD simulations of all charge states (oxidized, one- and two-electron reduced), histidine remains "trapped" in between the quinone methoxy groups with hydrogen bonds, thereby preventing the delivery of the proton to doubly-reduced UQ, and resulting in the formation of UQH^- with a single proton donated by tyrosine (Movie S3). We note this alternative arrangement of histidine bound to methoxy groups of UQ is distinguished from the *Sus scrofa* complex I multiscale simulations, where histidine relocates itself within the timescales of classical and QM/MM simulations to form the hydrogen bonding with the ketonic group of UQ (see Discussion). This difference in part can arise from the modeling procedure and/or due to the subtle differences in *Yarrowia lipolytica* and *Sus scrofa* complex I active sites. Nevertheless, the obtained conformation of neutral histidine bound in between the two methoxy groups represents an alternative binding mode of UQ in the UQ tunnel.

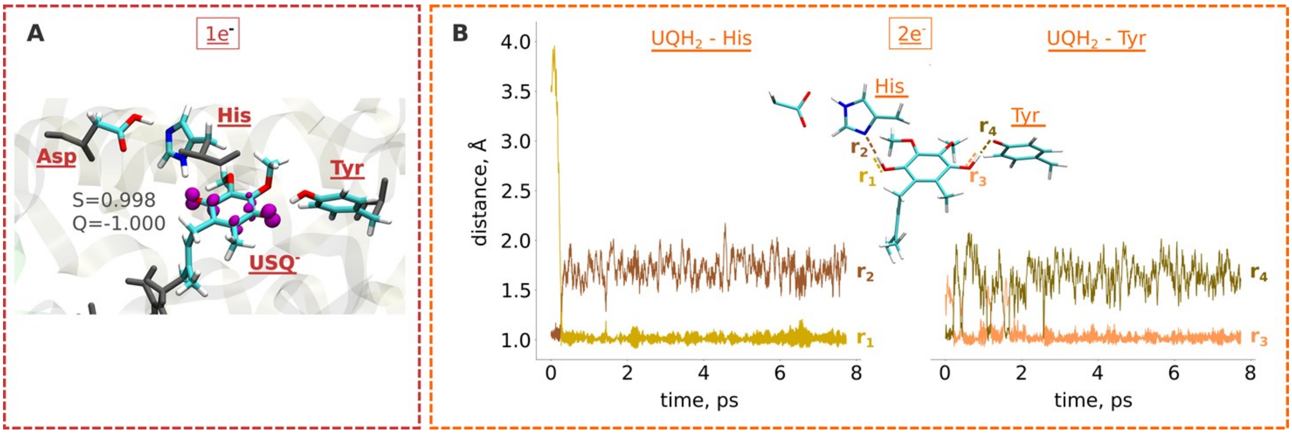

Fig. S1. (**A**) Spin density distribution (shown as magenta surface, isovalue 0.01) from the USQ^- model system obtained from the 1000th fs of the unbiased QM/MM MD simulation. Captions indicate total charge (Q) and spin (S), calculated with Mulliken population analysis. (**B**) Protonation dynamics in the two-electron reduced state observed in our unbiased QM/MM MD simulations. The distances between the quinone and His (left panel) and Tyr (right panel) are shown. The distance notations are introduced in the inset.

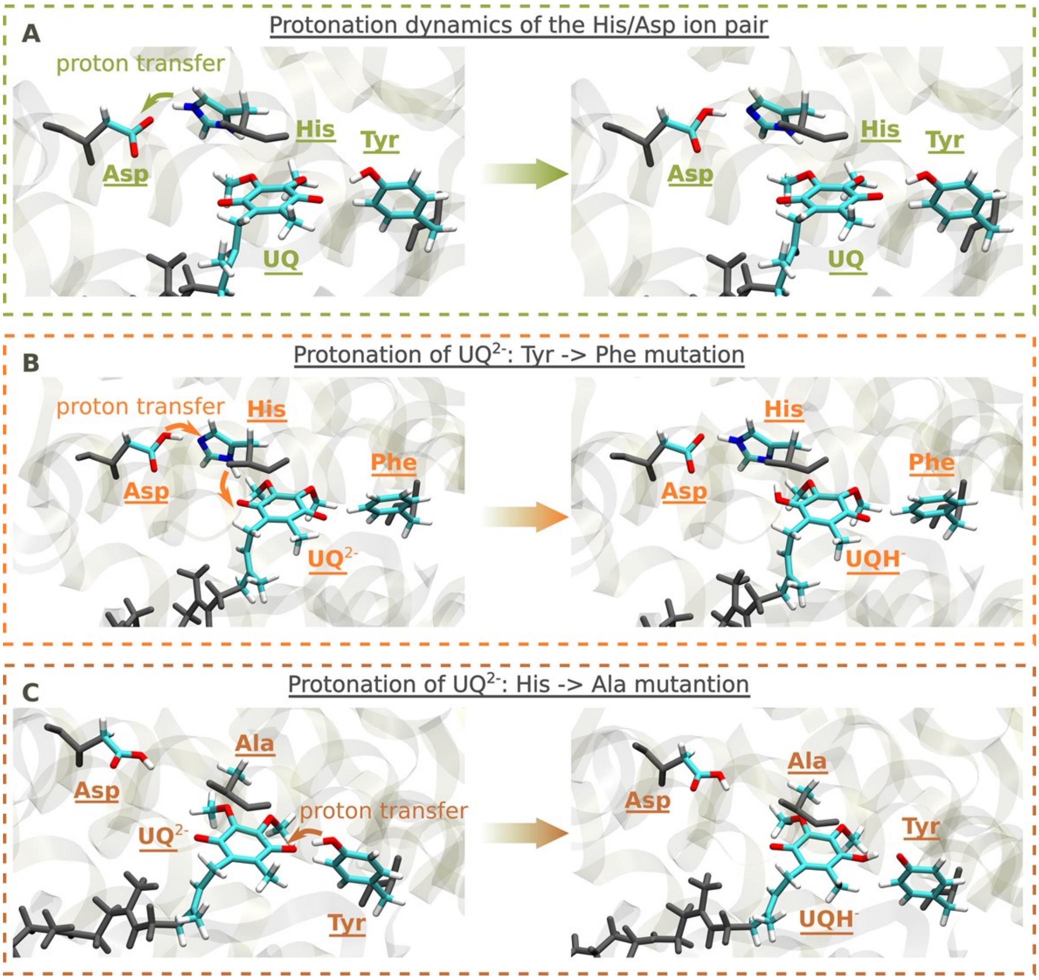

Fig. S2. Spontaneous protonation dynamics in unbiased QM/MM simulations: (**A**) Internal proton transfer within the His/Asp pair during the QM/MM minimization of oxidized state (and also one-electron reduced state, see main text). (**B**) Proton transfer from the His/Asp pair to UQ during unbiased QM/MM MD simulations of two-electron reduced system, where Tyr is mutated to Phe. (**C**) Protonation of doubly-reduced ubiquinone from Tyr during unbiased QM/MM MD simulation, where His is mutated to Ala.

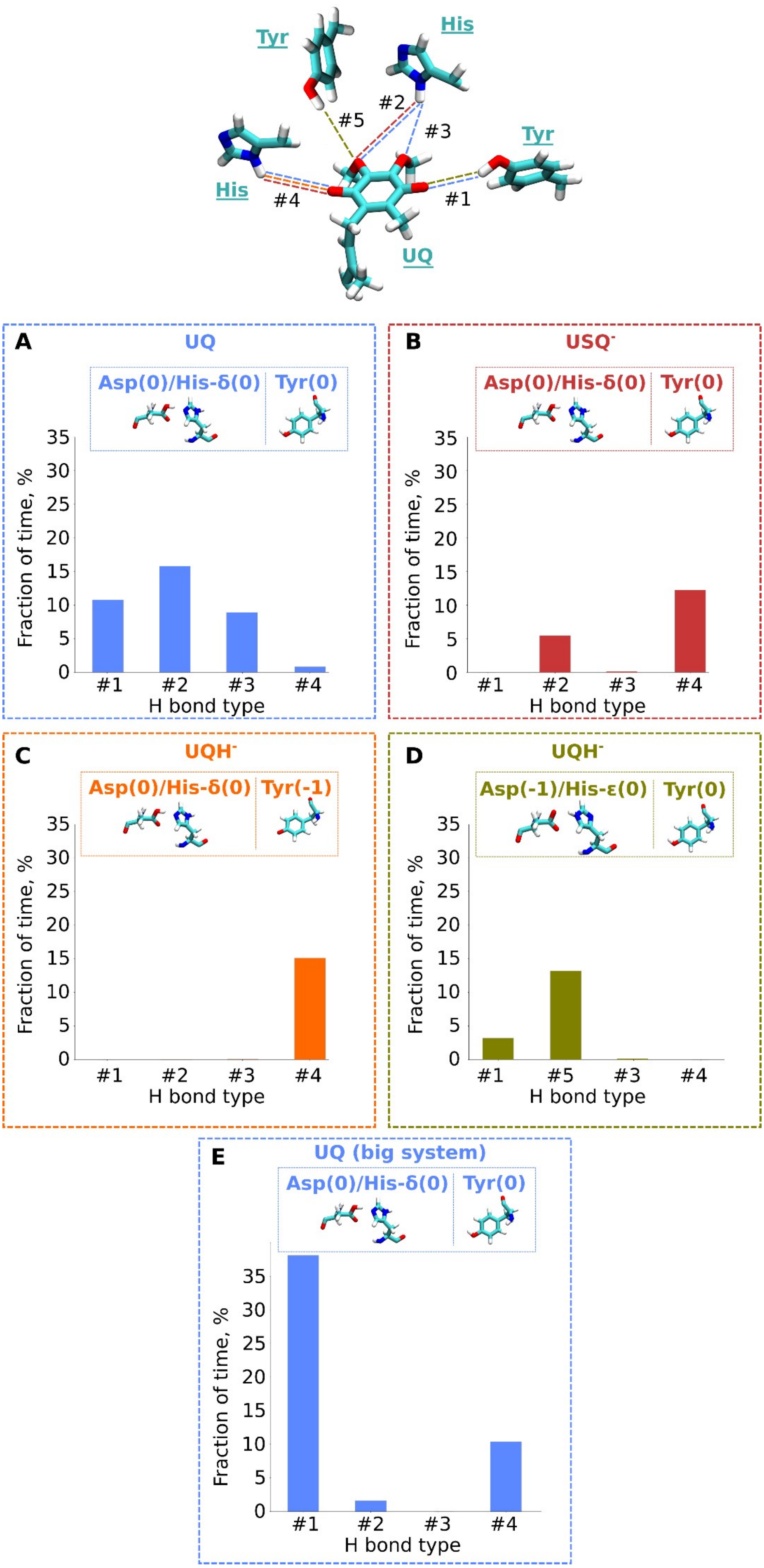
Tyr
His
#2
#5
#3
Tyr
His
#4
#1
UQ
A
UQ
Asp(0)/His-δ(0)
Tyr(0)
Fraction of time, %
H bond type
#1
#2
#3
#4
B
USQ-
Asp(0)/His-δ(0)
Tyr(0)
Fraction of time, %
H bond type
#1
#2
#3
#4
C
UQH-
Asp(0)/His-δ(0)
Tyr(-1)
Fraction of time, %
H bond type
#1
#2
#3
#4
D
UQH-
Asp(-1)/His-ε(0)
Tyr(0)
Fraction of time, %
H bond type
#1
#5
#3
#4
E
UQ (big system)
Asp(0)/His-δ(0)
Tyr(0)
Fraction of time, %
H bond type
#1
#2
#3
#4
0
5
10
15
20
25
30
35

Fig. S3. Formation of hydrogen bonds by the UQ head group with the conserved residues at the UQ binding site 1, obtained from classical unbiased MD simulations. Top panel illustrates the types of hydrogen bonds listed on the x-axis of panels (**A**) – (**E**). Colors of the dashed lines denote the hydrogen bonds that are prevalent (exist more than 2.5% of the simulated time) in the respective setups (**A**) – (**E**). Insets on panels (**A**) - (**E**) indicate the modeled protonation states of the titratable residues, as well as the UQ charge state. The data in panels (**A**) – (**D**) is from MD simulations of *Yarrowia lipolytica* complex I (PDBId: 7O71, Table S4, see also computational methods), whereas panel (**E**) illustrates the results from a large-scale model system of complex I from *Sus Scrofa* (PDBId: 7V2C; setup 2, Table S4; see also methods).

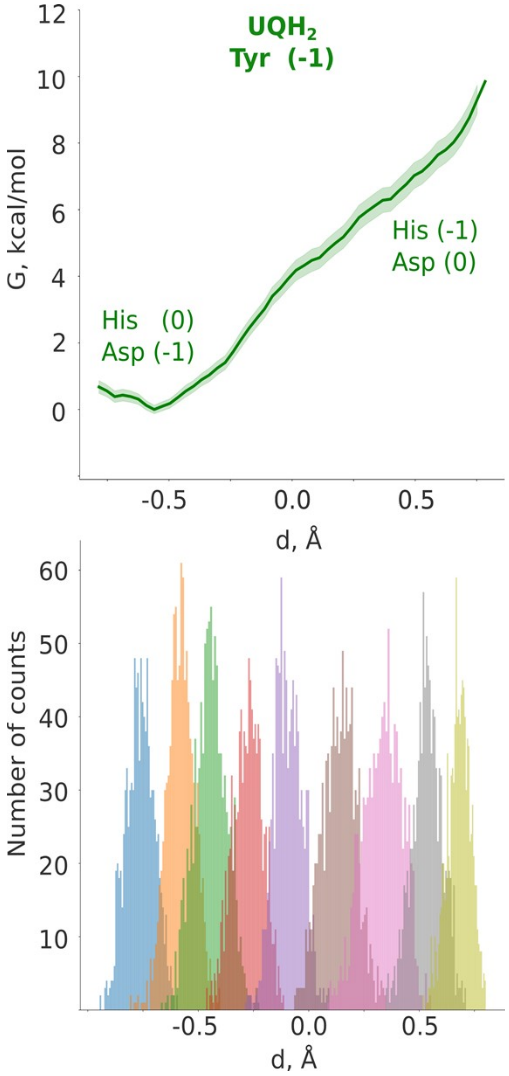

Fig. S4. QM/MM US free energy calculations of proton transfer from His to Asp in two-electron reduced case. Free energy profile (top panel), and US histograms of the reaction coordinate (bottom panel) sampled from the simulation windows are shown.

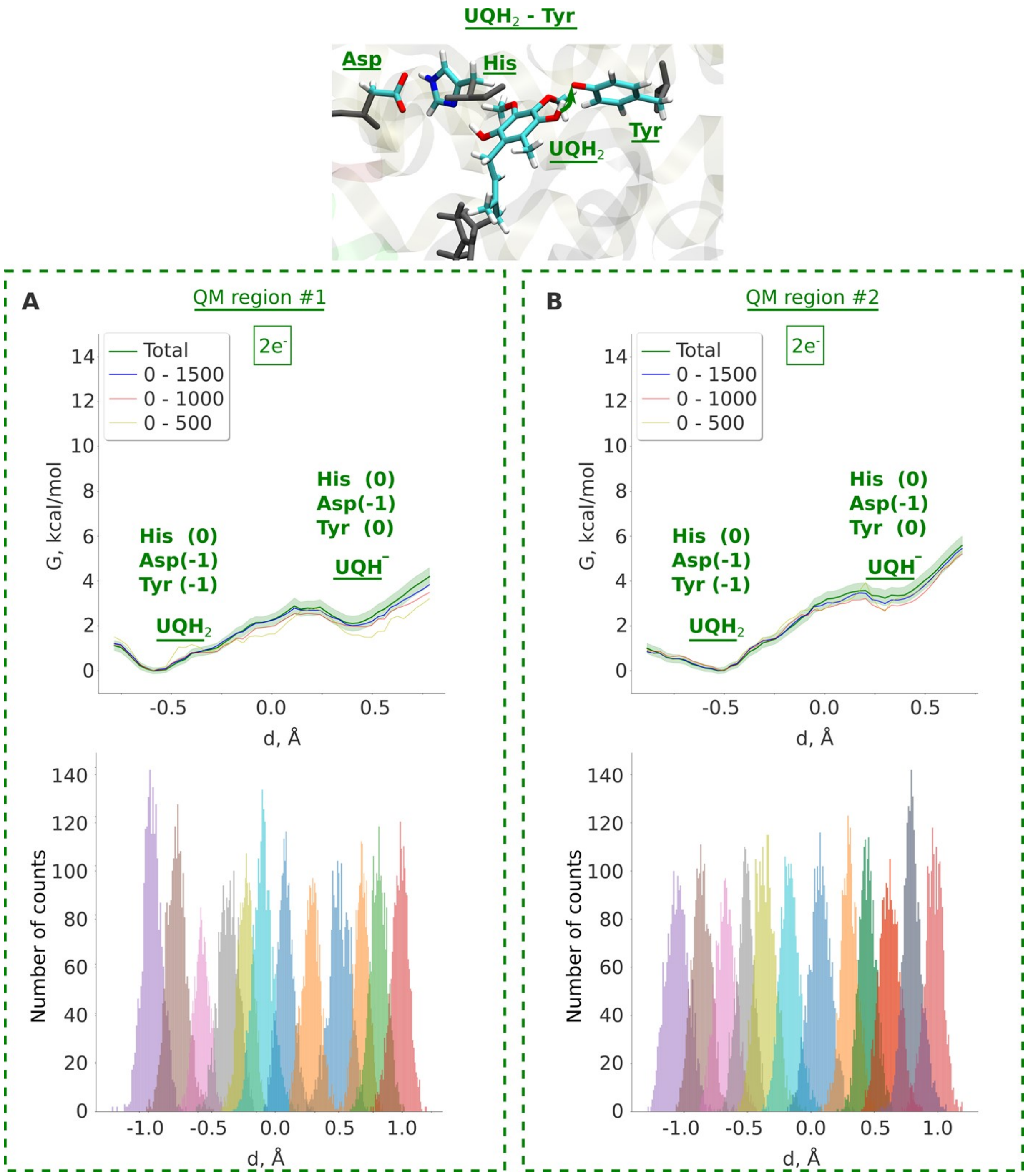

Fig. S5. Comparison between the results from QM/MM umbrella sampling simulations of proton transfer from UQH_2 to anionic Tyr in different QM regions (see Table S1): (**A**) QM region #1, results are also shown in Fig. 2B (right curve) of the main text. (**B**) Larger QM region (QM region #2). The captions to each energy state indicate the charge of the respective surrounding residues. Top panels show free energy profiles, and the bottom ones – US histograms of the reaction coordinate in the respective simulation windows. Arrow in the top inset indicates the biasing direction of the proton (UQH_2 to anionic Tyr) in the umbrella sampling simulations (see also Computational Methods).

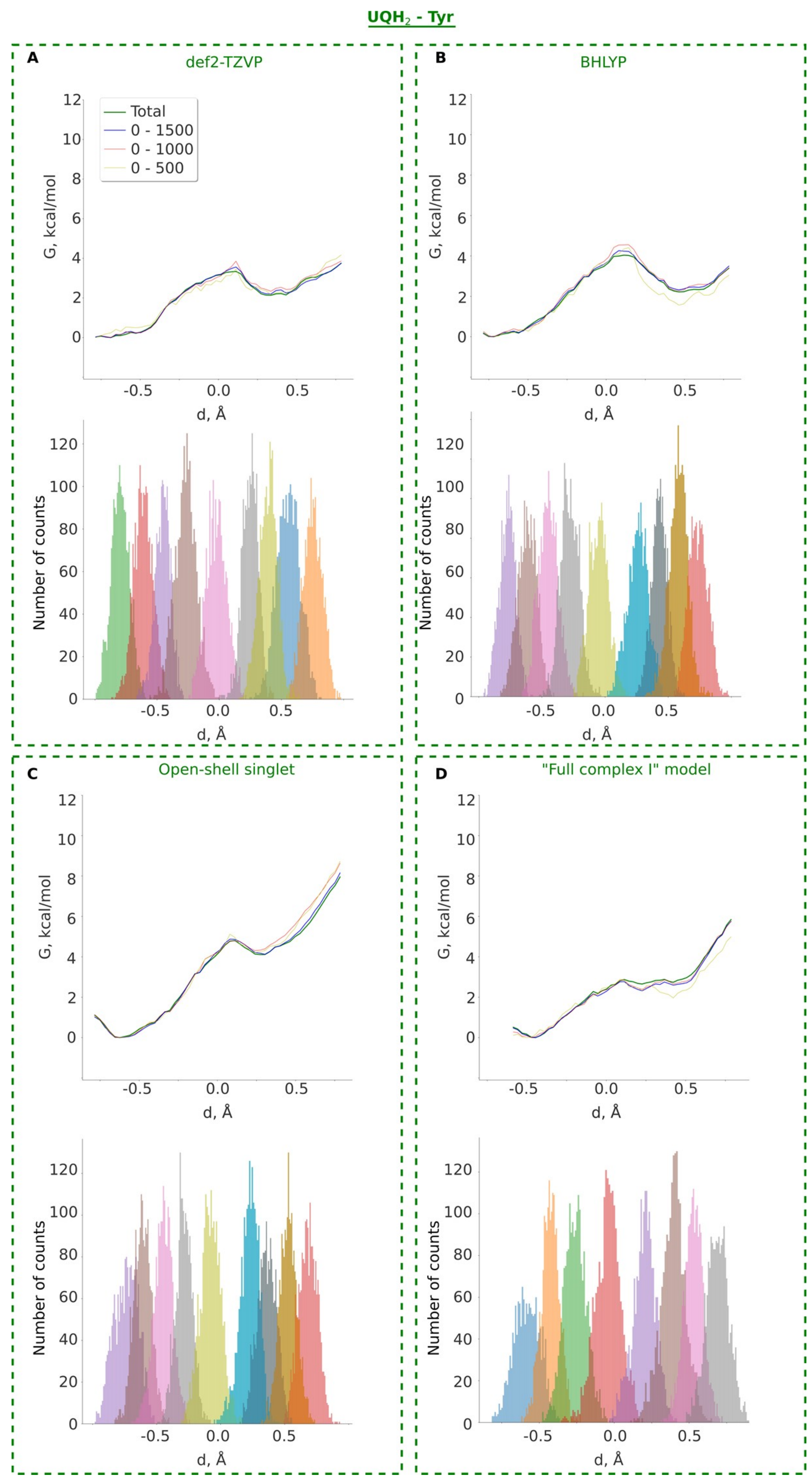
UQH$_2$ - Tyr
A
def2-TZVP
B
BHLYP
C
Open-shell singlet
D
"Full complex I" model
Total
0 - 1500
0 - 1000
0 - 500
G, kcal/mol
d, Å
Number of counts

Fig. S6. Free energy profile (top) and biased histograms (bottom) of proton transfer from UQH_2 to anionic Tyr using: (**A**) split-valence triple-zeta def2-TZVP basis set with the B3LYP functional, (**B**) BHLYP functional with the def2-SVP basis set, (**C**) unrestricted density functional theory (open-shell singlet state) with B3LYP functional and def2-SVP basis set, (**D**) B3LYP functional with def2-SVP basis set on the model system comprising all core and accessory subunits of mitochondrial respiratory complex I. Simulation setups in (**A**) - (**C**) were constructed from the truncated model system from *Y. lipolytica* (PDBId:7O71), while results in (**D**) were obtained from full complex I structure from *S. scrofa* (PDBId:7V2C, see also Table S2). Partial contributions to the free energy profiles from the first 500 fs, 1000 fs, and 1500 fs are shown as brown, red, and blue lines, respectively, highlighting convergence.

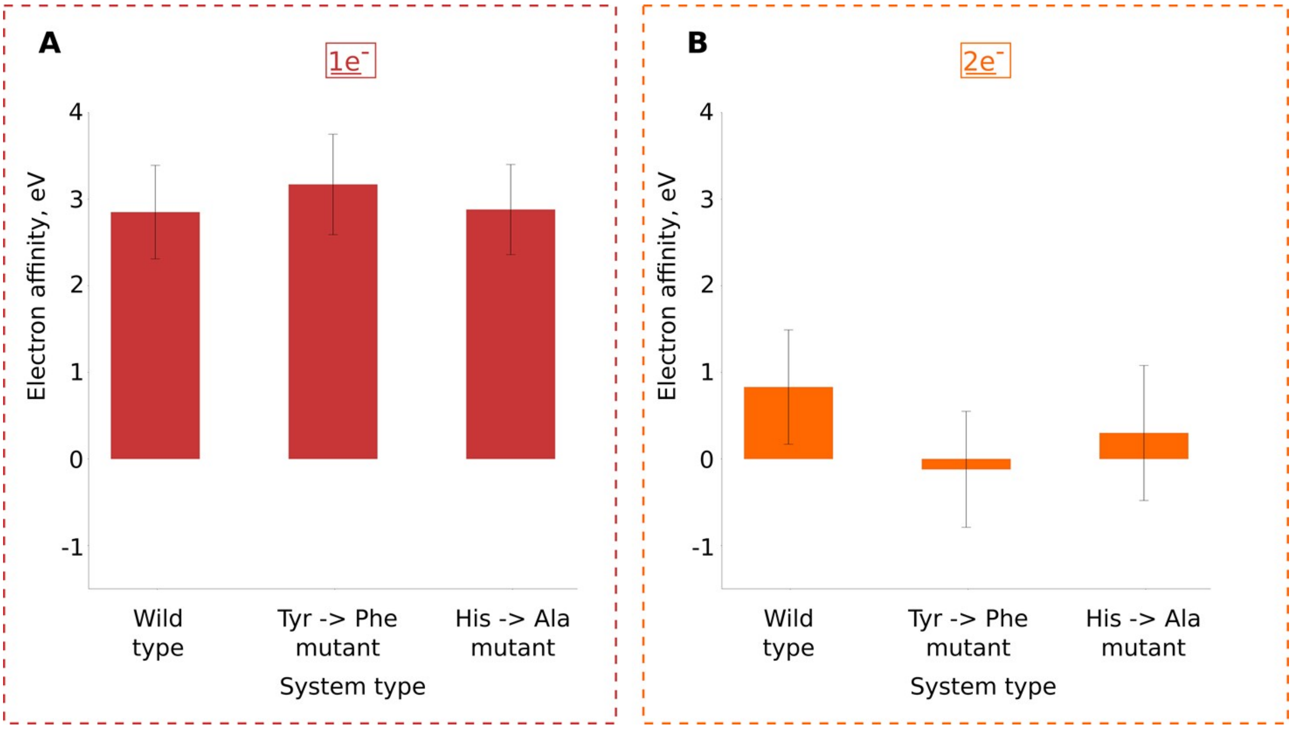

Fig. S7. Electron affinities for the first (1e$^-$) and second (2e$^-$) electron transfers, calculated based on the QM/MM energies (see Computational Methods). Proton-coupled electron transfer upon adding one and two electrons to the system is illustrated in Fig. S2 B,C.

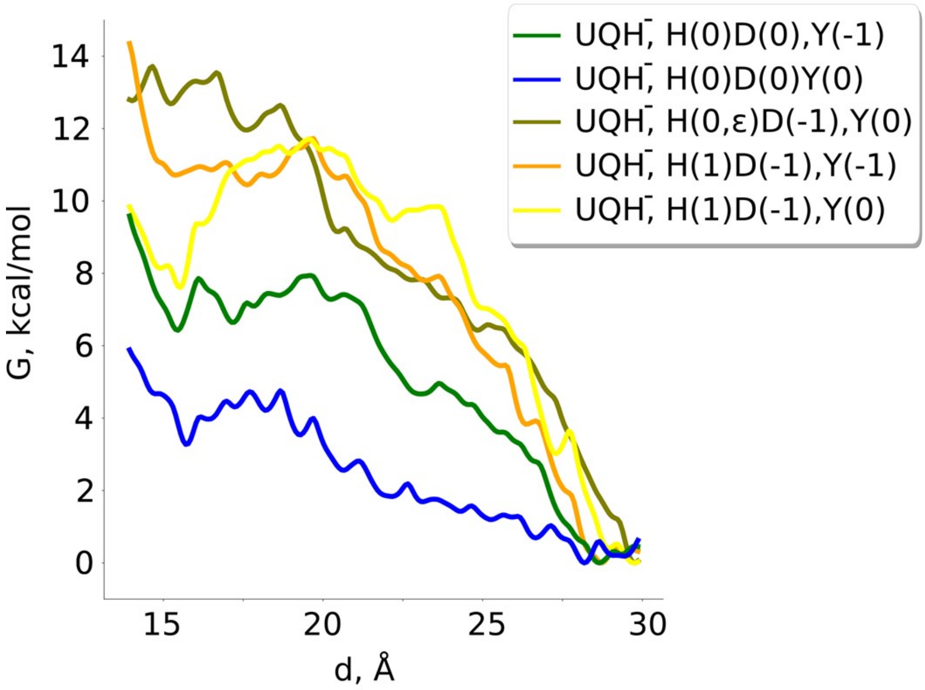

Fig. S8. Classical free energy profiles of the anionic ubiquinol species (UQH^-) in the binding chamber of *Yarrowia lipolytica* respiratory complex I with different charge states of the adjacent titratable residues: neutral His/Asp pair with anionic Tyr (green line); neutral His/Asp pair with neutral Tyr (blue line); neutral His with negatively charged Asp and neutral Tyr (olive-green line); His/Asp ion pair with anionic Tyr (orange line); His/Asp ion pair with neutral Tyr (yellow line).

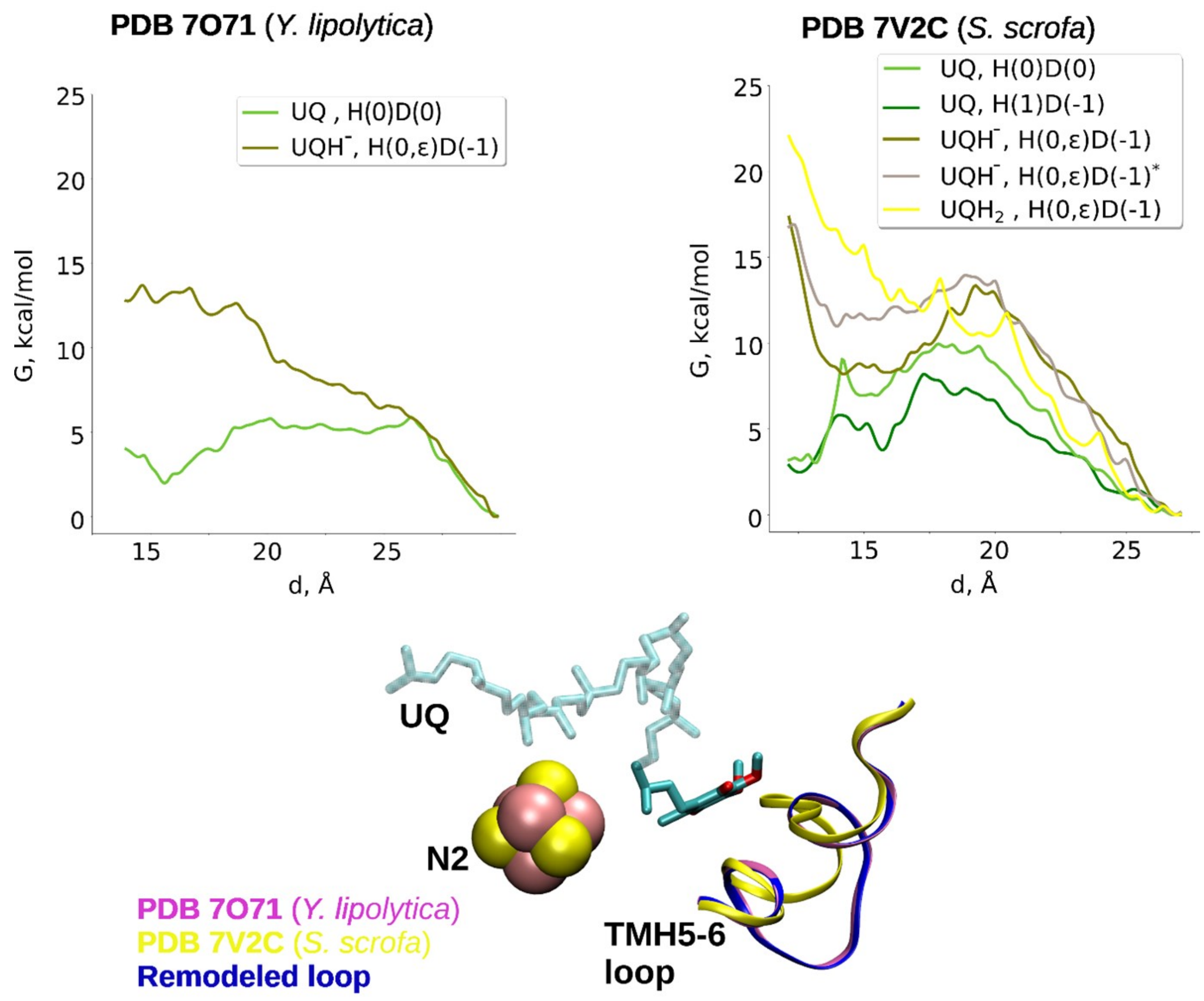

Fig. S9. Comparison of classical PMF (free energy) profiles of the ubiquinone species in the binding tunnel of respiratory complex I from *Yarrowia lipolytica* (top, left) and *Sus Scrofa* (top, right). The redox/protonation states of ubiquinone (UQ), and histidine (H) and aspartic acid (D) are mentioned. All PMF profiles are obtained in the structural conformations, except the grey PMF profile (*) in the top right panel, which is obtained when the TMH5-6 loop of ND1 in the porcine complex I structure is remodeled in the conformation observed in the *Yarrowia* complex I structure (see lower panel, see also Computational Methods). Note, in UQH^-, the tyrosine residue is neutral, but in UQH_2 it is anionic. The PMF profiles of *Yarrowia* complex I (left panel) show energetics of oxidized ubiquinone (UQ) with neutral His/Asp pair (light green) and UQH^- with neutral His (epsilon), negatively charged Asp and neutral Tyr (olive green). Similarly, the PMF profiles of porcine complex I (right panel) show energetics of UQ with His/Asp pair in neutral state (light green); UQ with His/Asp pair in ion-pair state (dark green); UQH^- with neutral His (epsilon), negatively charged Asp and neutral Tyr (olive green); UQH^- with neutral His (epsilon), negatively charged Asp and neutral Tyr with TMH5-6 loop remodeled (grey) and UQH_2 with neutral His (epsilon), negatively charged Asp and anionic Tyr (yellow). See also Fig. 4.

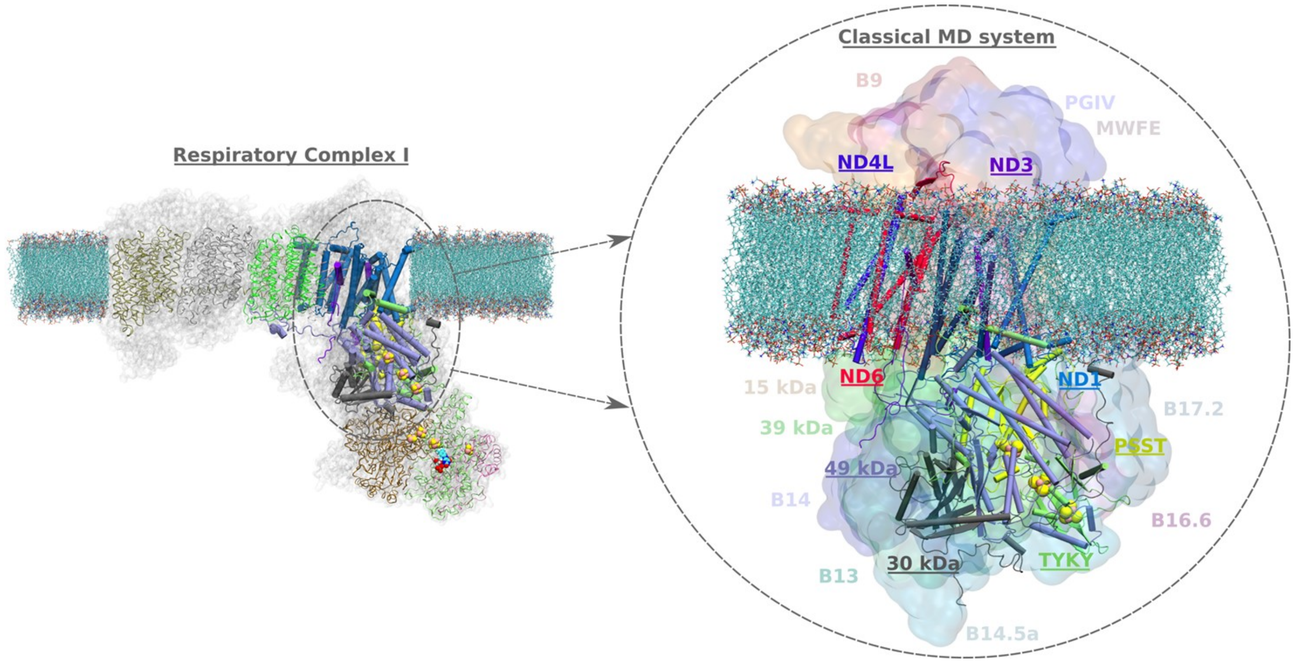

Fig. S10. Model system for classical MD simulations (right panel) constructed from the high-resolution structure of mitochondrial complex I from *Yarrowia lipolytica* (left panel, PDB ID: 7O71) [1]. Cartoon representation in the right panel depicts the core subunits included in the model system. The accessory subunits are shown in surface representation.

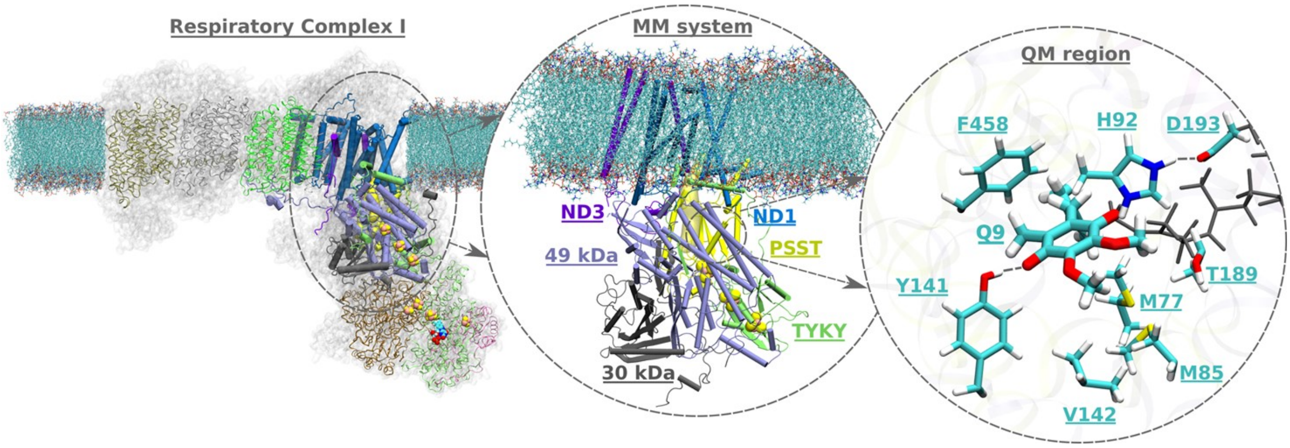

Fig. S11. Model system for hybrid QM/MM simulations of high-resolution structure of mitochondrial complex I from *Sus scrofa* (PDB ID: 7V2C [2]). Middle panel depicts the MM system consisting of 6 core subunits shown in respective colors: ND1, ND3, 49 kDa, 30 kDa, PSST, and TYKY. Water box and ions, which are also included in the MM region are not shown for clarity. Right panel illustrates protein residues and quinone head group included in the QM region (QM region #1, Table S1). Hydrophobic isoprenyl tail of UQ included in the MM region is shown in grey licorice representation.

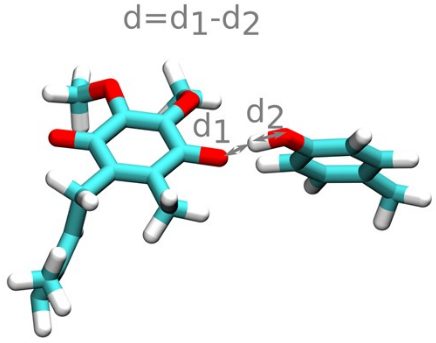

Fig. S12. An example reaction coordinate used in the QM/MM MD umbrella sampling simulations.

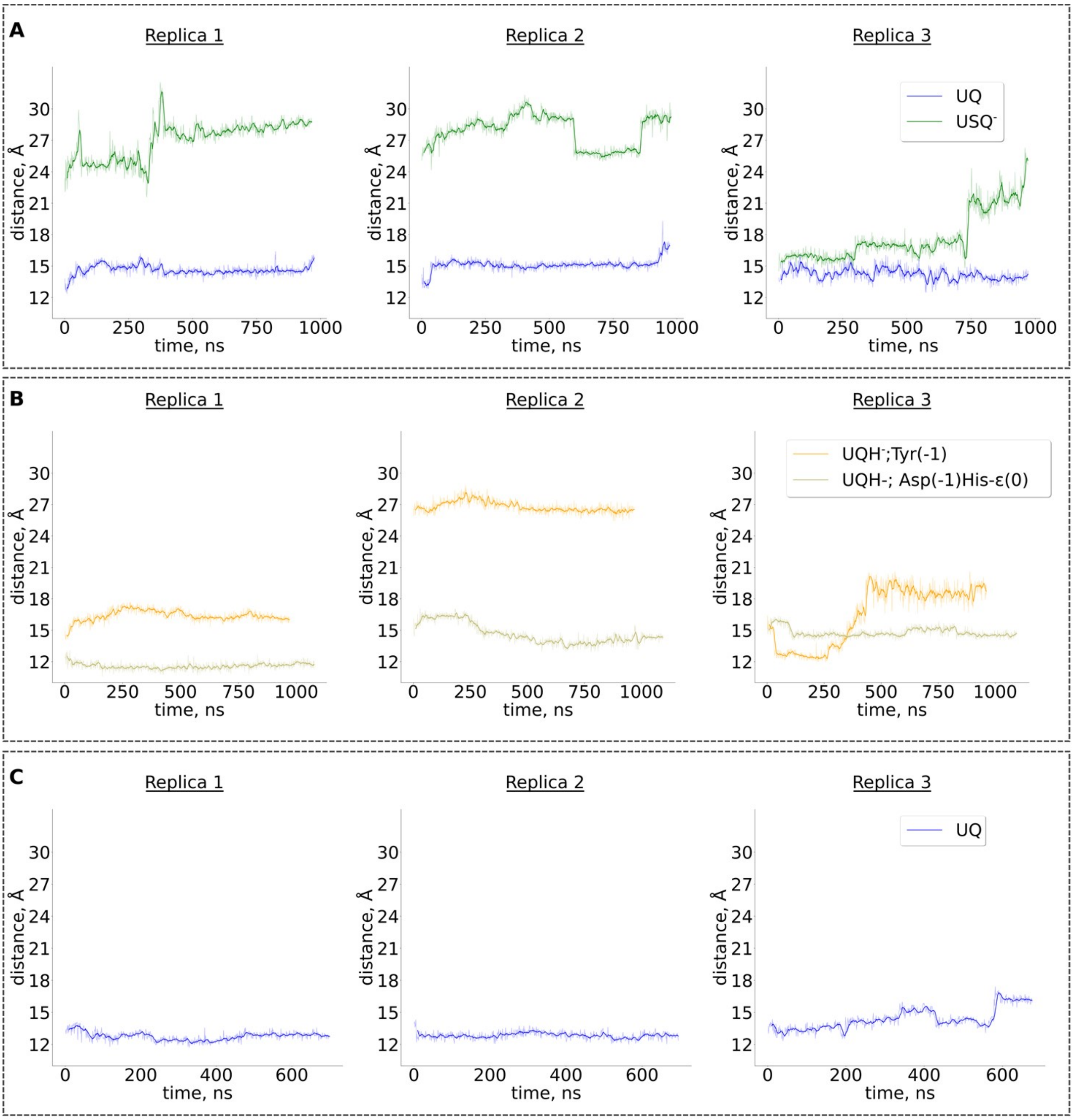

Fig. S13. (**A-C**) Time series of the UQ-N2 distances (Fig. 3, main text) from unbiased classical MD simulations for different charge states of the UQ species: (**A**) oxidised ubiquinone (UQ, blue) and anionic ubisemiquinone (USQ^-, green). (**B**) anionic ubiquinol (UQH^-) with deprotonated Tyr (orange curve), and with deprotonated Asp and neutral His with the ε nitrogen protonated (brown curve). Results in (**A**, **B**) are obtained from simulations of the truncated model system of respiratory complex I from *Y. lipolytica* (Fig. S10, see also Table S4). (**C**) UQ-N2 distances in the full-scale model of respiratory complex I from *Sus scrofa* where ubiquinone is modelled in the oxidised state (UQ), see Table S4.

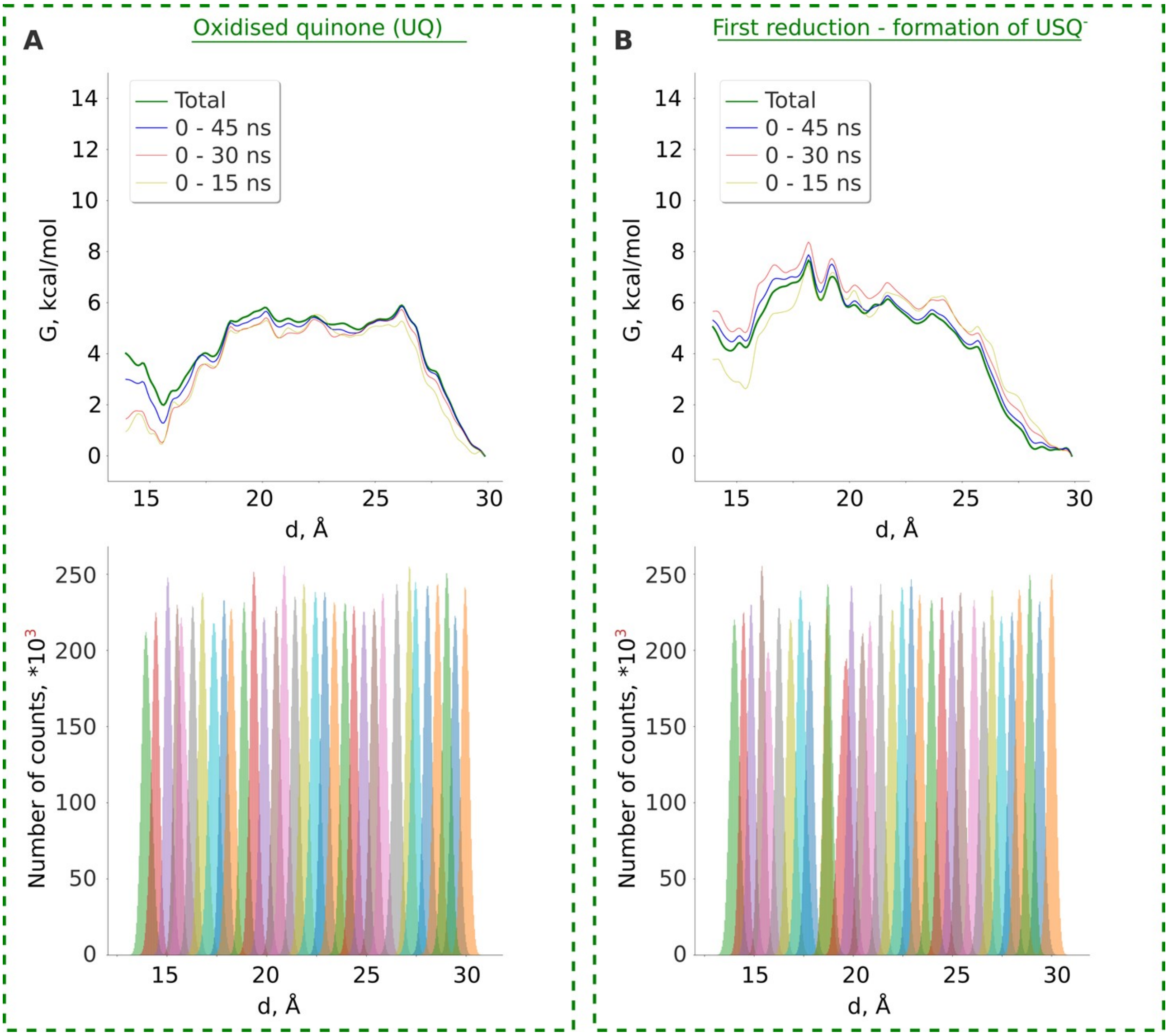

Fig. S14. Classical US free energy calculations of the oxidised UQ (**A**) and anionic USQ^- (**B**) in the quinone binding chamber. Top panels show free energy profiles, while the bottom panels depict the respective histograms. Partial contributions to the total free energy profile from the first 15, 30, and 45 ns are shown with light brown, red, and blue lines, respectively.

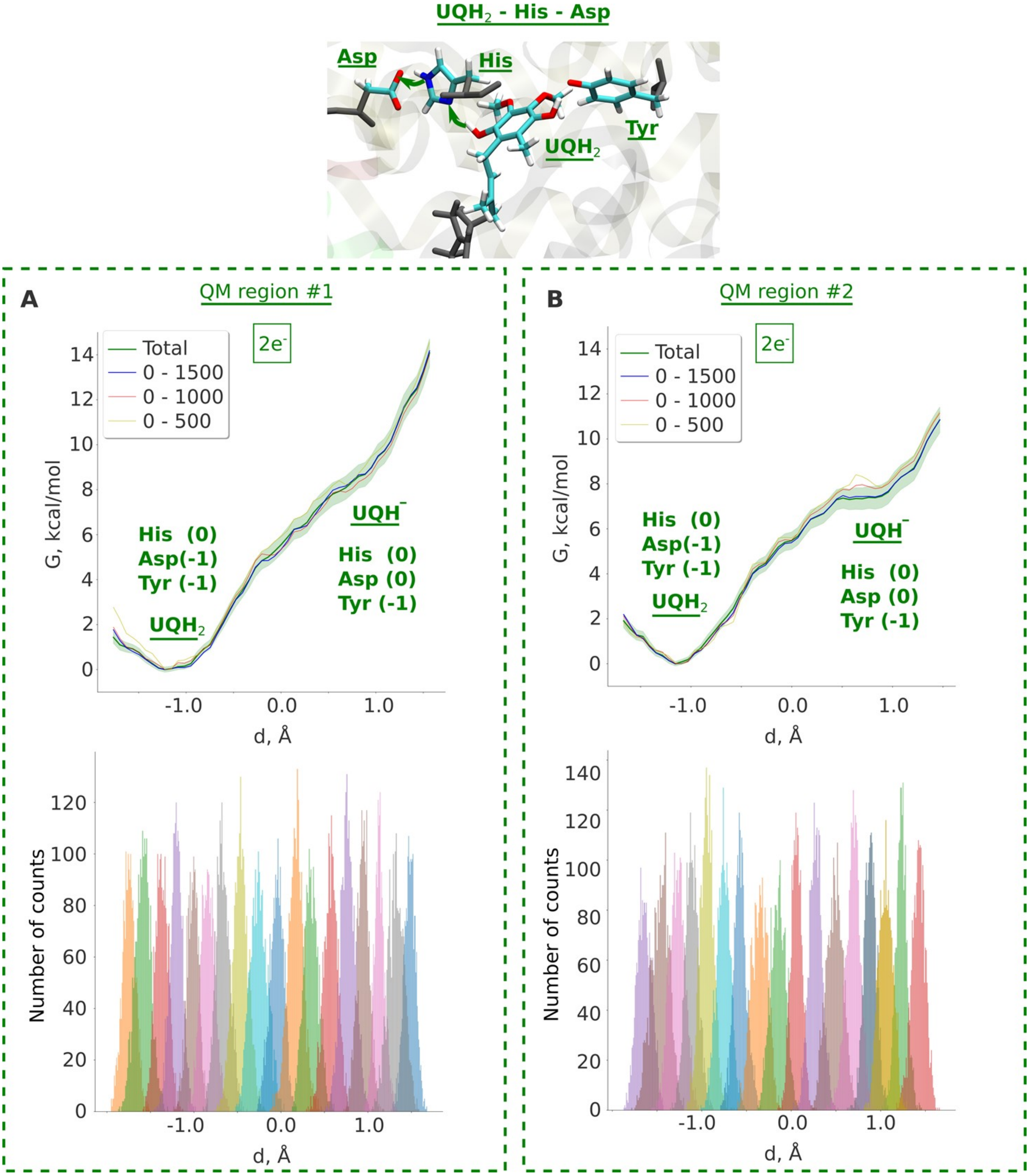

Fig. S15. Free energy profiles obtained from QM/MM umbrella sampling simulations of the proton transfer from UQH_2 to the anionic His/Asp pair, where both hydrogen bonds (UQH_2..His and His...Asp) are included in the reaction coordinate (see inset). QM region #1 (**A**) and QM region #2 (**B**) (for QM region notations, see Table S1). Charges of the surrounding residues are depicted in captions to the respective energy states. Bottom panels show normalized distributions of the reaction coordinate in simulation windows. Arrows in the top inset indicates the biasing direction of the proton (UQH_2 to anionic His/Asp pair) in the umbrella sampling simulations (see also methods).

Movie S1. Conformational rearrangement of His during the unbiased QM/MM MD simulation of a one-electron reduced state. The trajectory length included in the movie is 2.0 ps.

Movie S2. Protonation dynamics of ubiquinone during the unbiased QM/MM MD simulation in a two-electron reduced state. The movie is rendered from a 2.0-ps trajectory.

Movie S3. Formation and chemical stability of the quinol anion in the two-electron reduced system where quinone methoxy groups were initially symmetrically arranged with respect to each other. (see supplementary text). The video includes a 200-steps QM/MM minimization, as well as the full 3.7-ps dataset of unbiased QM/MM MD (see Table S2).

Table S1. QM regions considered in this work.

QM region number	Starting structure (PDBId)	Number of atoms (excluding link hydrogens)	Residues included in the QM region
1	7V2C [2]	122	^{49kD}Asp193, ^{49kD}His92, ^{49kD}Tyr141, ^{49kD}Met185, ^{49kD}Phe458, ^{49kD}Thr189, ^{49kD}Val142, ^{PSST}M77, Q9 head group (QM/MM bond along C11-C12)
1.1	7V2C	131	^{49kD}Asp193, ^{49kD}His92, ^{49kD}Tyr141, ^{49kD}Met185, ^{49kD}Phe458, ^{49kD}Thr189, ^{49kD}Val142, ^{PSST}M77, Q9 head group (QM/MM bond along C11-C12), 3 water molecules aligned from the high-resolution structure from *Bos taurus* (PDB ID: 8Q48) [3] (^{49kD}Wat606, ^{49kD}Wat643, ^{49kD}Wat695)
2	7V2C	150	^{49kD}Asp193, ^{49kD}His92, ^{49kD}Tyr141, ^{49kD}Met185, ^{49kD}Phe458, ^{49kD}Thr189, ^{49kD}Val142, **^{49kD}Lys404**, **^{49kD}Asn182**, **^{49kD}Ile456**, ^{PSST}M77, Q9 head group (QM/MM bond along C11-C12)
3	7O71	162	^{49kD}Asp196, ^{49kD}His95 ^{49kD}Tyr144, ^{49kD}Met188, ^{49kD}Phe461, ^{49kD}Thr189, ^{49kD}Ser192, ^{49kD}Phe203, ^{49kD}Met195, ^{49kD}Val145, ^{49kD}Val97, ^{PSST}Met91, ^{PSST}Ala87, Q9 head group (QM/MM bond along C11-C12), 6 water molecules populating the QM region in the unbiased classical MD simulation‡
4	7O71	161	^{49kD}Asp193, ^{49kD}His92, ^{49kD}Tyr141, ^{49kD}Met185, ^{49kD}Phe458, ^{49kD}Thr189, ^{49kD}Val142, ^{PSST}M77, Q9 head group (QM/MM bond along C11-C12), 13 water molecules populating the QM region in the unbiased classical MD simulation$^{\#}$

‡ the starting configuration for the QM/MM setups was taken from the classical MD snapshot after 300 ns of the production run (Table S4, setup 1, replica1; see also Table S2)

$^{\#}$ the starting configuration for the QM/MM setups was taken from the classical MD snapshot after 300 ns of the production run (Table S4, setup 2, replica1; see also Table S2)

Table S2. Wild-type QM/MM MD simulation setups constructed from the structures (PDBIds 7V2C and 7O71) [1,2].

Initial structure (PDBId)	Equilibration protocol[1]	Extra electrons	QM/MM min	QM/MM MD	QM region (see Tabl. S1)	Umbrella sampling	Density functional/ Basis set
7V2C	Std	0e-	+	2.0 ps	1	-	B3LYP/def2-SVP
7V2C	Std	1e-	+	2.0 ps	1	UQ-Tyr141	B3LYP/def2-SVP
7V2C	Std	2e-	+	7.8 ps	1	UQ-Tyr141	B3LYP/def2-SVP, B3LYP/def2-TZVP, BHLYP/def2-SVP
						UQ-His92<br>His92-Asp193<br>UQ-Tyr141<br>with constraints on His92(NE2-HE2)	B3LYP/def2-SVP
7V2C	Std	2e-	+	1.0 ps	1	UQ-Tyr141	B3LYP/def2-SVP[2]
7V2C[3]	Std	2e-	+	2.5 ps	4	UQ-Tyr141	B3LYP/def2-SVP
7V2C	Std	2e-	+	7.8 ps	2	UQ-Tyr141	B3LYP/def2-SVP
7V2C[#]	Std	0e-	+	2.1 ps	1.1	-	B3LYP/def2-SVP
7V2C[#]	Std	2e-	+	1.4 ps	1.1	-	B3LYP/def2-SVP
7V2C[&]	Std	2e-	+	1.2 ps	1.1	UQ-Tyr	B3LYP/def

							2-SVP
7V2C*	Std + Add	0e-	+	3.7 ps	1	-	B3LYP/def 2-SVP
7V2C*	Std + Add	2e-	+	3.7 ps	1	-	B3LYP/def 2-SVP
7O71‡	Std	0e-	+	1.2 ps	3	-	B3LYP/def 2-SVP
7O71‡	Std	2e-	+	1.64 ps	3	-	B3LYP/def 2-SVP

[1] "Std" stands for "standard" equilibration protocol, while "Std+Add" introduces an additional 10-ns equilibration stage with constraints on protein backbone, see the "Computational methods" section.

[2]this setup was simulated in the framework of the unrestricted density-functional theory (open-shell singlet state)

[3]the model system included all core and accessory subunits of respiratory complex I; the starting point was the last snapshot from classical MD production run (Table S4, setup 2, see also methods)

*one of two ubiquinone methoxy groups (CH_3-) was rotated to achieve symmetrical conformation of the UQ head group

#the setup was created by aligning 3 water molecules (^{49kD}Wat606, ^{49kD}Wat643, ^{49kD}Wat695) from [3] (PDB ID: 8Q48), located in the vicinity of the QM region, to the 7V2C structure

& the setup was created by aligning 3 water molecules (^{49kD}Wat606, ^{49kD}Wat643, ^{49kD}Wat695) from [3] (PDB ID: 8Q48), located in the vicinity of the QM region, to the 7V2C structure. The starting snapshot for QM/MM runs was taken after 1 ps of unbiased QM/MM MD of the "Std" 7V2C setup with 2 additional electrons

‡ the starting configuration for the QM/MM setups was taken from the classical MD snapshot after 300 ns of the production run

Table S3. QM/MM MD simulation setups with introduced mutations (Tyr/Phe and His/Ala) constructed from the structure (PDBId: 7V2C).

Initial structure (PDBId)	Equilibration protocol[1]	Extra electrons	QM/MM min	QM/MM MD	QM region (see Tabl. S1)	Umbrella sampling	Basis set
**Mutant: Tyr144 -> Phe**							
7V2C	Std	0	+	2.75 ps	1[2]	-	def2-SVP
7V2C	Std	1e-	+	1.97 ps	1[2]	-	def2-SVP
7V2C	Std	2e-	+	3.4 ps	1[2]	-	def2-SVP
**Mutant: His92 -> Ala**							
7V2C	Std	0	+	2.94 ps	1[3]	-	def2-SVP
7V2C	Std	1e-	+	1.5 ps	1[3]	-	def2-SVP
7V2C	Std	2e-	+	2.92 ps	1[3]	-	def2-SVP

[1] "Std" stands for "standard" equilibration protocol, while "Std+Add" introduces an additional 10-ns equilibration stage with constraints on protein backbone, see the "Computational methods" section.

[2] QM region is the same as #1 (see Tabl. S1), but with the mutation of Tyr144 to phenylalanine

[3] QM region is the same as #1 (see Tabl. S1), but with the mutation of His92 to alanine

Table S4. Classical MD simulation setups performed in the present work. In all simulations of PDB 7O71, the UQ head group is modeled based on resolved position of UQ in PDB 7V2C.

PdbID	# of atoms	Setup #	Protonation states			Ubiquinone charge state	Unbiased MD production simulation (time, ns)			US
			His[1]	Asp	Tyr		Replica 1	Replica 2	Replica 3	
7O71	~365,000	1.1	0 (δ)	0	0	UQ	~975 ns	~972 ns	~972 ns	+
		1.2				USQ^-	~966 ns	~977 ns	~971 ns	+
		1.3				UQH^-	-	-	-	+
		1.4	+1	-1	0	UQ	-	-	-	+
		1.5				USQ^-	-	-	-	+
		1.6				UQH^-	-	-	-	+
		1.7	0 (δ)	0	-1	UQH^-	~971 ns	~970 ns	~962 ns	+
		1.8	0 (ε)	-1	0	UQH^-	~1078 ns	~1095 ns	~1095 ns	+
		1.9	+1	-1	-1	UQH^-	-	-	-	+
		1.10								
		1.11								
7V2C	~ 1.6 M	2.1	+1	-1	0	UQ	~701 ns	~696 ns	~677 ns	-
		2.2	0 (δ)	0	0	UQ	~787 ns	~801 ns	~719 ns	-
		2.3	0 (ε)	-	0	UQH^-	~791 ns	~791 ns	~797 ns	-
		2.4	0 (ε)	-	-	UQH_2	~758 ns	~785 ns	~793 ns	-
	~435,000	3.1	+1	-1	0	UQ	-	-	-	+
		3.2	0 (ε)	-1	0	UQH^-	-	-	-	+
		3.3#	0 (ε)	-1	0	UQH^-	-	-	-	+
		3.4	0 (δ)	0	0	UQ	-	-	-	+
		3.5	0 (ε)	-1	-1	UQH_2	-	-	-	+

[1]δ and ε describe the tautomeric state of neutral histidine (proton position on the δ and ε nitrogen, respectively)

#TMH5-6 of the ND1 subunit was remodelled to adapt the conformation from *Yarrowia* complex I structure (PDB ID 7O71; see methods)

References

[1] K. Parey et al., *High-Resolution Structure and Dynamics of Mitochondrial Complex I—Insights into the Proton Pumping Mechanism*, Science Advances **7**, eabj3221 (2021).

[2] J. Gu, T. Liu, R. Guo, L. Zhang, and M. Yang, *The Coupling Mechanism of Mammalian Mitochondrial Complex I*, Nature Structural & Molecular Biology **29**, 172 (2022).

[3] D. N. Grba, J. J. Wright, Z. Yin, W. Fisher, and J. Hirst, *Molecular Mechanism of the Ischemia-Induced Regulatory Switch in Mammalian Complex I*, Science **384**, 1247 (2024).
